# Pharmacokinetics and Pharmacodynamics of Remimazolam for Procedural Sedation in Children and Adolescents

**DOI:** 10.1097/ALN.0000000000005560

**Published:** 2025-05-12

**Authors:** Pieter J. Colin, Lynn H. Bichajian, Valentin R. Curt, Jeroen V. Koomen, Thomas Stöhr, Michel M. R. F. Struys, Keira P. Mason

**Affiliations:** 2 Department of Anesthesiology, University of Groningen, University Medical Center Groningen, Groningen, The Netherlands.; 3 Eagle Pharmaceuticals Inc., Woodcliff Lake, New Jersey.; 4 Eagle Pharmaceuticals Inc., Woodcliff Lake, New Jersey.; 5 Department of Anesthesiology, University of Groningen, University Medical Center Groningen, Groningen, The Netherlands.; 6 Paion Pharma GmbH, Aachen, Germany.; 7 Department of Anesthesiology, University of Groningen, University Medical Center Groningen, Groningen, The Netherlands; Department of Basic and Applied Medical Sciences, Ghent University, Ghent, Belgium.; 8 Department of Anesthesiology, Boston Children’s Hospital, Boston, Massachusetts.

## Abstract

**Background:**

Remimazolam is not approved for use in pediatric patients. The pharmacokinetics of remimazolam have been reported to be similar to those of adult patients after scaling for body size. This article reports on the pharmacokinetics and pharmacodynamics of pediatric patients aged 6 to 18 yr and a subsequent model-based optimization of the used dosing regimen.

**Methods:**

Thirty-one patients were included in the trial and stratified across four treatment arms: bolus administration, infusion, bolus plus fentanyl, or infusion plus fentanyl. The University of Michigan (Ann Arbor, Michigan) Sedation Scale (UMSS) was used to assess the depth of sedation. Blood samples were drawn to measure the concentrations of remimazolam and its metabolite CNS7054. Population pharmacokinetic pharmacodynamic modeling was performed in NONMEM (GloboMax LLC, USA).

**Results:**

A population pharmacokinetic model was developed for remimazolam and CNS7054. The elimination clearance of remimazolam was 0.70 l · min^–1^ · 70 kg^–1^. A proportional odds model combined with a simplified Minto model described the observed UMSS well. The EC50 of remimazolam for a UMSS score of 3 or greater was 777 ng · ml^–1^ in the absence of fentanyl, and decreased to 655, 533, and 287 ng/ml for concomitant fentanyl steady state concentrations of 1, 2, or 4 ng · ml^–1^, respectively. Simulations confirmed that the studied dosing regimen resulted in 9.2 to 22.0% of patients not reaching a UMSS score of 3 or greater at the end of the induction. Model-based optimization resulted in higher per-kilogram dosages and the removal of the maximum allowable dose. Simulations indicated that the percentage of patients achieving a UMSS score of 3 or greater can be expected to be high (88 to 97%).

**Conclusions:**

This study has shown that the pharmacokinetics of remimazolam are likely different between children 6 yr or older and adults (after correcting for size). In addition, the exposure–response relationship shows that to effectively use remimazolam for procedural sedation in children 6 yr or older, the dosing regimen should be modified to allow for higher remimazolam exposures.

## Abstract 2

This study evaluated the efficacy, safety, and pharmacokinetics of remimazolam for intravenous sedation in 31 patients between 6 and 18 yr of age undergoing diagnostic or therapeutic procedures. Patients were stratified across four treatment arms (bolus, infusion, bolus plus fentanyl, or infusion plus fentanyl), and the University of Michigan Sedation Scale was used to assess the depth of sedation. Remimazolam pharmacokinetics and the exposure–response relationship in children between 6 and 18 yr of age appear to differ from those reported in adults. Simulations based on these data suggest that for effective sedation in children 6 yr old and older, the dosing regimen should allow for higher remimazolam exposures.

## Editor’s Perspective

**What We Already Know about This Topic**

- Remimazolam, a short-acting benzodiazepine that is indicated for procedural sedation, is not approved for use in pediatric patients

**What This Article Tells Us That Is New**

- This study evaluated the efficacy, safety, and pharmacokinetics of remimazolam for intravenous sedation in 31 patients between 6 and 18 yr of age undergoing diagnostic or therapeutic procedures
- Patients were stratified across four treatment arms (bolus, infusion, bolus plus fentanyl, or infusion plus fentanyl), and the University of Michigan Sedation Scale was used to assess the depth of sedation
- Remimazolam pharmacokinetics and the exposure–response relationship in children between 6 and 18 yr of age appear to differ from those reported in adults
- Simulations based on these data suggest that for effective sedation in children 6 yr old and older, the dosing regimen should allow for higher remimazolam exposures

Remimazolam (Byfavo) is a short-acting benzodiazepine that is indicated for procedural sedation (European Union, United States, China, and South Korea) and general anesthesia (European Union, Japan, and South Korea) in adults.^1–3^ The approved U.S. Food and Drug Administration (Silver Spring, Maryland) dosing regimen for adult procedural sedation consists of an intravenous induction bolus of 5 mg with top-up boluses of 2.5 mg to maintain appropriate sedation levels. In the European Union, the approved dosing regimen offers two possible options, one that includes fentanyl and one that does not: 5 mg with 2.5 mg top-up boluses in the presence of opioids or 7 mg with 2.5 mg top-up boluses in the absence of opioids.

The rapid onset of action and relatively rapid recovery profile may offer advantages for procedural sedation in children. There are very few reports of the use of remimazolam describing its efficacy, safety, and pharmacokinetics in the pediatric population.^4,5^ Gao *et al.*5 showed that the pharmacokinetics of remimazolam and its metabolite, CNS7054, could be described by a three- and two-compartment model, respectively, in pediatric patients aged 2 to 6 yr undergoing general anesthesia with sevoflurane. After correcting for body size, using body weight–based scaling of pharmacokinetic parameters with the West–Brown–Enquist^6^ allometric exponents, the pharmacokinetic profiles of remimazolam and CNS7054 were considered similar to those of adults.^5^ Moreover, cardiovascular and respiratory safety were deemed acceptable.^4^

The exposure–response relationship and the interplay with the coadministration of opioids has not been studied in detail in the pediatric population. Therefore, in this study, we aimed to describe the pharmacokinetics and pharmacodynamics of remimazolam in the pediatric patient population aged 6 yr and older undergoing procedural sedation. Furthermore, using model simulations, we aimed to determine the dosing regimen that could achieve the required depth of sedation, as determined by the University of Michigan (Ann Arbor, Michigan) Sedation Scale^7^ (UMSS), in pediatric patients aged 6 yr and older undergoing procedural sedation.

## Materials and Methods

In 2021, a clinical trial (EudraCT, 2020-004118-37; ClinicalTrials.gov, NCT04851717; sponsor No. CNS7056-026/DA10030) was initiated to assess the efficacy of intravenous remimazolam in inducing and achieving targeted sedation levels in pediatric patients undergoing diagnostic and/or therapeutic procedures. This trial was a multinational trial, of which parts 1 and 2 are conducted in the United States and part 3 will be conducted in Europe. Advarra (Columbia, Maryland) approved the study in the United States on April 23, 2021. This trial is part of the Pediatric Investigational Plan and the Pediatric Study Plan that have been developed to comply with the pediatric regulations of the European Medicines Agency (Amsterdam, The Netherlands) and the U.S. Food and Drug Administration, respectively.

### Inclusion and Exclusion of Patients

For this analysis, patients 6 yr or older and 18 yr or younger were recruited in the United States. They were part of the first cohort of a phase 2/3, prospective, open-label trial evaluating the efficacy, safety, and pharmacokinetics of remimazolam for intravenous sedation in pediatric patients undergoing diagnostic and/or therapeutic procedures.

Eligibility criteria allowed for the inclusion of a diverse pediatric population (American Society of Anesthesiologists [Schaumburg, Illinois] Physical Status ranging from I to III) presenting for elective diagnostic or therapeutic procedures requiring sedation of less than 2 h duration. Inclusion criteria required a negative pregnancy test for females who had reached menses, the use of medically accepted contraception during the trial period for females of childbearing potential, and anticipated spontaneous breathing during the procedure.

Exclusion criteria included any procedure requiring endotracheal tube or laryngeal mask insertion; emergency procedures; any airway or craniofacial anomalies that could compromise emergency airway rescue or predispose to airway obstruction during spontaneous ventilation; hypersensitivity to any of the ingredients in the drug product; history of paradoxical reactions to benzodiazepines; obstructive sleep apnea; active respiratory, cardiac, or hepatic failure; or active neuromuscular disease. Written informed consent and assent (if applicable) were obtained from all parents/patients.

### Study Design

Patients were stratified across dosing modalities (bolus *vs*. infusion), different depth-of-sedation targets (UMSS score 1 to 2 *vs*. UMSS score 2 to 3, defined as Supplemental Digital Content table 1, https://links.lww.com/ALN/E8) and presence or absence of fentanyl coadministration, resulting in eight treatment arms. The modality, sedation target, and decision to use fentanyl were left to the investigators’ discretion. Fentanyl was to be used only for analgesia during painful procedures. When fentanyl was used, it was administered 2 to 5 min before the first remimazolam bolus. The dosing of fentanyl bolus and supplements were 50 µg and 25 µg, respectively, for patients 12 or older and 18 yr or younger and 1 µg · kg^–1^ and 0.5 µg · kg^–1^, respectively, for patients 6 or older to younger than 12 yr. Remimazolam dosing across the eight treatment arms according to the initial protocol is summarized in table 1. The minimum allowable interdose interval was at least 3 min.

**Table 1.** Initial Dosing Protocol for Remimazolam

	Bolus Group	Bolus + Fentanyl Group	Infusion Group	Infusion + Fentanyl Group
Target UMSS scores 1–2				
Initial bolus (µg · kg^-1^)	60 (max. 7.0 mg)	45 (max. 5.0 mg)	—	—
Initial infusion (µg · kg^-1^ · min^-1^)	—	—	2.5 (max. 7.0)	2.0 (max. 5.0)
Top-up bolus (µg · kg^-1^)	30	20	30	20
Target UMSS scores, 2–3				
Initial bolus (µg · kg^-1^)	125 (max. 7.0 mg)	100 (max. 5.0 mg)	—	—
Initial infusion (µg · kg^-1^ · min^-1^)	—	—	5.0 (max. 15)	3.5 (max. 10)
Top-up bolus (µg · kg^-1^)	60	50	60	50

All dose calculations should be rounded to two decimal places for patients weighing < 25 kg and to one decimal place for patients weighing ≥ 25 kg.

Max., maximum; UMSS: University of Michigan Sedation Scale.

### Study Procedures and Measurement of Drug Effect

During the study, a board-certified pediatric anesthesiologist was responsible for the remimazolam delivery and all aspects of sedation management, following the American Society of Anesthesiologists guidelines for moderate sedation.^8^ Only nitrous oxide or intravenous fentanyl was permitted to facilitate intravenous access or procedural sedation conditions, respectively. The UMSS^7^ was used to assess the level of sedation. Depth of sedation measured by the UMSS ranged from 0 (awake/alert) to 4 (unarousable). UMSS was scored at baseline and was evaluated every minute from the time of the first dose of remimazolam until the start of the procedure. The procedure was started when the targeted depth of sedation was reached. Subsequently, UMSS was measured every 3 min until termination of the procedure and every minute thereafter until the patient was arousable, then every 5 min until the patient met discharge criteria.

Rescue sedative medication was administered when the desired level of sedation was not achieved or maintained during any 15-min window, and top-up bolus doses were given every 3 min. The procedure was continued with any other sedative appropriate for pediatric use at the investigator’s discretion (*e.g.*, midazolam, propofol, dexmedetomidine).

### Pharmacokinetic Sampling and Analysis of Blood Samples

Remimazolam and CNS7054 concentrations were determined in venous plasma samples, which were taken at various timepoints. Blood samples (volume of sample, 300 µl) were collected 2 min (±15 s) after the start of remimazolam, immediately before the procedure start, before the second top-up dose, before the fourth top-up dose, at the end of the procedure, 5 min after the procedure, 30 min after the procedure, and 1.5 h after the procedure. Blood was collected in K_2_EDTA 4-ml tubes and stored at 4°C or in a wet ice bath until they were centrifuged (within 40 min after collection) at 2,000*g* at 4°C for 10 min. The remimazolam plasma samples were stored at –20°C until analysis at the bioanalytical laboratory as described elsewhere.^9^ In short, remimazolam and metabolite CNS7054 concentrations were extracted from plasma using solid-phase extraction and analyzed using ultra-high-performance liquid chromatography–mass spectrometry at Aptuit (Verona, Italy). Deuterium-labeled remimazolam and CNS7054 were used as internal standards. The lower and upper limits of quantification were 2 ng/ml and 2,000 ng/ml (remimazolam) and 20 ng/ml and 20,000 ng/ml (CNS7054), with a within-run precision of 4.6% or less and 1.9% or less for remimazolam and CNS7054, respectively.

### Population Pharmacokinetic Modeling

A population pharmacokinetic model was developed using the plasma remimazolam and plasma CNS7054 concentration data. Model parameters were estimated using the first-order conditional estimation with interaction algorithm in NONMEM (Version 7.4; GloboMax LLC, USA). The “tidyverse” package^10^ (version 1.3.1) in R (version 4.0.5, R Foundation for Statistical Computing, Vienna, Austria) was used to graphically assess the goodness-of-fit of the candidate models.

As a starting point for the joint remimazolam–CNS7054 pharmacokinetic model development, we evaluated a three-compartment remimazolam model linked to a one-compartment CNS7054 model. To avoid overparameterization, we fixed the fraction metabolized to 1.00, assuming that remimazolam is 100% metabolized to CNS7054, and assuming a molar conversion ratio CNS7054 to remimazolam of 1.033 (Pubchem compound identification numbers for remimazolam and CNS7054 are 9867812 and 46941174, respectively). All pharmacokinetic parameters were scaled *a priori* to weight according to allometric theory (0.75 and 1.00 for elimination clearance and volume of distribution parameters, respectively).^6^

Interindividual variability on the population parameters was assumed to be log-normally distributed. Residual unexplained variability was modeled using additive, proportional, or additive and proportional error models.

Inclusion of covariates in the model was driven by graphical evaluation of the relationship between random effects and the covariates. Covariates evaluated for inclusion in the model were age, sex, and concomitant fentanyl administration.

### Population Pharmacodynamic Modeling

The “individual pharmacokinetic parameter approach”^11^ was applied to develop the pharmacodynamic models. A proportional odds logistic regression model was fitted to the UMSS observations using the second-order conditional estimation with interaction algorithm in NONMEM (version 7.4). The proportional odds logistic regression model was parameterized such that the parameters in the model expressed the effect-site concentrations achieving a 50% probability for a particular UMSS category k (EC50_k_ with k ∈ {0, 1, 2, 3} as UMSS score of 4 was not observed). The ordered categorical nature of the UMSS was preserved by modeling successive EC50 values as the sum of the EC50 values for the lower ranking scores according to equations 1 and 2 where EC50_k_ and ΔEC50_k_ are parameters estimated from the data.


$$\begin{array}{l} EC50_{2}=EC50_{1}+\Delta EC50_{2} \end{array}$$ (1)

$$\begin{array}{l} EC50_{3}=EC50_{2}+\Delta EC50_{3} \end{array}$$ (2)

To account for the potential interaction between remimazolam and fentanyl, we first predicted fentanyl arterial plasma concentrations using the model by Ginsberg *et al.*^12^ based on the patient’s weight and individual fentanyl dosing history. Subsequently, a potential delay between fentanyl-induced effects and the time course of the predicted fentanyl arterial plasma concentrations was handled by the addition of an effect–compartment model. The predicted fentanyl effect-site concentration served as the input for a simplified interaction model described by Minto *et al.*^13^ assuming additive interaction between remimazolam and fentanyl as shown in equations 3 to 5.


$$\begin{array}{l} U_{RMZ,k}=\frac{C_{e,RMZ}}{EC50_{RMZ,k}} \end{array}$$ (3)

$$\begin{array}{l} U_{FENT,k}=\frac{C_{e,FENT}}{EC50_{FENT,k}} \end{array}$$ (4)

$$\begin{array}{l} E_{k}=\frac{{\left(U_{RMZ,k}+U_{FENT,k}\right)}^{\gamma }}{1+{\left(U_{RMZ,k}+U_{FENT,k}\right)}^{\gamma }} \end{array}$$ (5)

In this model, U_RMZ,k_ and U_FENT,k_ are the normalized effect-site concentrations for remimazolam and fentanyl for UMSS score k (with k ∈ {0, 1, 2, 3}), which is obtained by dividing the predicted effect-site concentrations of remimazolam (Ce_RMZ_) and fentanyl (Ce_FENT_) by the respective estimated effect-site concentrations achieving the half-maximum effect for remimazolam (EC50_RMZ,k_) and fentanyl (EC50_FENT,k_) on UMSS score k. The resulting combined drug effect of remimazolam and fentanyl on UMSS score k (E_k_) is a nonlinear function of the sum of the normalized effect-site concentrations for both drugs with γ denoting the steepness of the nonlinear relationship. The probabilities for the individual UMSS scores can be directly derived from E_k_, where P(Y = 0) = 1 – E_1_, P(Y = 1) = E_1_ – E_2_, P(Y = 2) = E_2_ – E_3_, and P(Y = 3) = E_3_. This parameterization assumes that P(UMSS = 0) = 100% at baseline.

In line with the work by Vellinga *et al.*,^9^ we tested a model for tolerance development, driven by competitive antagonism by CNS7054. For this, the normalized concentration for CNS7054 (U_CNS7054_) was the ratio of the predicted plasma concentration of CNS7054 (C_CNS7054_) and the plasma concentration achieving half-maximum competitive inhibition (IC50_CNS7054_), as seen in equation 6. The combined drug effect of remimazolam, fentanyl, and CNS7054 on UMSS score k (E_k_) was modeled according to equation 7.


$$\begin{array}{l} U_{CNS7054}=\frac{C_{CNS7054}}{IC50_{CNS7054}} \end{array}$$ (6)

$$\begin{array}{l} E_{k}=\frac{{\left(U_{RMZ,k}+U_{FENT,k}\right)}^{\gamma }}{1+{\left(U_{RMZ,k}+U_{FENT,k}+U_{CNS7054}\right)}^{\gamma }} \end{array}$$ (7)

Once the structural model was identified, covariates were screened for inclusion in the model. Covariates considered were weight, age, sex, concomitant fentanyl administration, and study procedure (bone marrow aspiration, endoscopy, lumbar punction, or other).

### Model Building and Evaluation

During model building candidate models were compared with likelihood ratio testing based on the NONMEM objective function value (OFV) at the 5% level of significance (decrease of 3.84 points in the OFV per 1 degree of freedom). Modifications to the models were accepted only if they resulted in a significant decrease in the OFV, demonstrated improvements in the goodness-of-fit plots, and were estimable with acceptable numeric stability (*i.e.*, condition number less than 500).

Internal model validation was based on prediction variance–corrected visual predictive checks.^14^ For all model parameters, the maximum likelihood estimate and the associated 95% CI derived from log-likelihood profiling are reported.

### Model-informed Dosing Regimen Optimization

To facilitate simulation, the final models were implemented in RxODE,^15^ and calculations were parallelized on an HP Z640 workstation with an Intel E5-2670 version 3 (2.30 GHz) 12-core processor.

Simulations to inform the pediatric dosing regimen were based on a virtual cohort of 5,000 pediatric patients. Virtual patients were 6 yr or older and younger than 18 yr, and 50% were girls. The Sumpter and Holford^16^ equation was used to simulate body weights based on sex and age (assuming 40 weeks’ gestational age), taking into account stochastic noise arising from unexplained between-subject variability.

Remimazolam and fentanyl (when applicable) concentration time profiles were simulated using the final population pharmacokinetic model presented in this paper and the model by Ginsberg *et al.*,^12^ respectively. Unexplained between-subject variability was included in the pharmacokinetic simulations for remimazolam but was not included in the simulations for fentanyl using the model by Ginsberg *et al.*^12^ The dosing regimen of fentanyl in the simulations consisted of (1) a fentanyl loading dose administered 4 min before the first remimazolam bolus, and (2) a maintenance dose 10 min after the first remimazolam bolus dose. Fentanyl doses were 50 µg and 25 µg, respectively, for patients 12 year or older and 18 yr or younger, and 1 µg · kg^–1^ and 0.5 µg · kg^–1^, respectively, for patients 6 yr or older to younger than 12 yr. Subsequently, the final pharmacokinetic–pharmacodynamic model for UMSS was used to simulate UMSS at 30-s intervals for a 30-min procedure, taking into account unexplained between-subject variability in the model parameters.

The simulations included titration to effect, where additional top-up doses were triggered when two consecutive UMSS values were less than 3, provided the time between top-up doses is at least 3 min. This was intended to resemble clinical practice, where an anesthesiologist titrates the dose to a sedation target (in this case, UMSS score 2 to 3).

The dosing regimen was optimized based on clinical feasibility and by varying (1) bolus dose, (2) top-up dose relative to the initial bolus dose, (3) maximum infusion rates, (4) number of dose escalations allowed during the induction phase, (5) maximum allowable total dose (“dose capping”) strategies, and (6) weight or age adjustments on the per-kilogram dose. The final optimized dosing regimen was selected based on the highest probability of target attainment.

## Results

### Revised Dosing Regimen

The original protocol planned for 40 patients to be included in the first age cohort (6 yr or older and younger than 18 yr). However, after enrolling the first 5 patients, the trial was briefly stopped due to a higher-than-expected incidence of hypotension adverse events. This was determined to be due to the use of propofol as rescue medication for inadequate sedation. Higher than expected need for rescue medication suggested that the proposed original dosing regimen of remimazolam was not suitable to achieve effective procedural sedation conditions. After careful evaluation of the data and patient responses, the maximum allowable infusion rates and the top-up dose were increased. The revised dosing regimen is detailed in table 2. The trial was restarted and continued to recruit an additional 26 patients before the trial was paused again due to the number of patients requiring rescue sedative medication to complete the procedure (9 of 31; table 3). During this second pause, while collaborating with the U.S. Food and Drug Administration to update the trial protocol, the trial sponsors decided to conduct this modeling and simulation analysis of the existing pharmacokinetic and pharmacodynamic data to support a more appropriate dosing scheme in pediatric patients.

**Table 2.** Revised Dosing Protocol for Remimazolam

	Bolus Group	Bolus + Fentanyl Group	Infusion Group	Infusion + Fentanyl Group
Target UMSS scores, 1–2				
Initial bolus (µg · kg^-1^)	60 (max. 7.0 mg)	45 (max. 5.0 mg)	—	—
Initial infusion (µg · kg^-1^ · min^-1^)	—	—	2.5 (max. 20 but not exceeding 1 mg · min^-1^)	2.0 (max. 20 but not exceeding 1 mg · min^-1^)
Top-up bolus (µg · kg^-1^)	30–60 (max. 7.0 mg)	20–45 (max. 5.0 mg)	30–60 (max. 7.0 mg)	20–45 (max. 5.0 mg)
Target UMSS scores, 2–3				
Initial bolus (µg · kg^-1^)	150 (max. 7.0 mg)	125 (max. 5.0 mg)	—	—
Initial infusion (µg · kg^-1^ · min^-1^)	—	—	7.5 (max. 20 but not exceeding 1 mg · min^-1^)	5.0 (max. 20 but not exceeding 1 mg · min^-1^)
Top-up bolus (µg · kg^-1^)	75–150 (max. 7.0 mg)	60–125 (max. 5.0 mg)	75–150 (max. 7.0 mg)	60–125 (max. 5.0 mg)

All dose calculations should be rounded to two decimal places for patients weighing < 25 kg and to one decimal place for patients weighing ≥ 25 kg.

Max., maximum; UMSS: University of Michigan Sedation Scale.

**Table 3.** Patient and Trial Characteristics

	Bolus Group	Bolus + Fentanyl Group	Infusion Group	Infusion + Fentanyl Group	All Patients
No. of patients	5	11	5	10	31
Age, yr	10.4 ± 4.0 [6–16]	12.9 ± 3.9 [6–17]	11.2 ± 2.3 [9–15]	13.1 ± 3.5 [8–18]	12.3 ± 3.6 [6–18]
Weight, kg	38.5 ± 15.3 [21–62]	59.9 ± 29.4 [20.5–106]	42.3 ± 11.0 [30.6–56.9]	50.4 ± 12.5 [26.8–70.4]	50.6 ± 21.2 [20.5–106]
Sex (female/male)	3/2	4/7	2/3	3/7	12/19
Type of procedure					
Bone marrow aspiration	—	4	—	—	4
Endoscopy	—	1	2	4	7
Lumbar punction	3	5	—	—	8
Other	2	1	3	6	12
No. of remimazolam top-up doses	4 [0–5]	6 [2–16]	3 [0–6]	2.5 [0–4]	4 [0–16]
No. of fentanyl doses	—	3 [1–6]	—	2 [1–5]	1 [0–6]
Use of rescue medication (yes/no)	1/4	2/9	3/2	3/7	9/22

Continuous variables are summarized by the mean ± SD and the range (between square brackets). The number of remimazolam top-up doses and the number of fentanyl doses are summarized as the median and the range (between square brackets).

### Demographics

Thirty-one patients were eligible and randomly allocated to the treatment arms. Since none of the investigators chose the lighter or mild sedation target (UMSS score 1 to 2), while the design allowed for a maximum of eight treatment arms, subjects were included in just four treatment arms. Five patients each were recruited in the bolus and the infusion group without concomitant fentanyl administration. Eleven and 10 patients, respectively, were recruited in the bolus and infusion group with concomitant fentanyl administration.

**Fig. 1.**
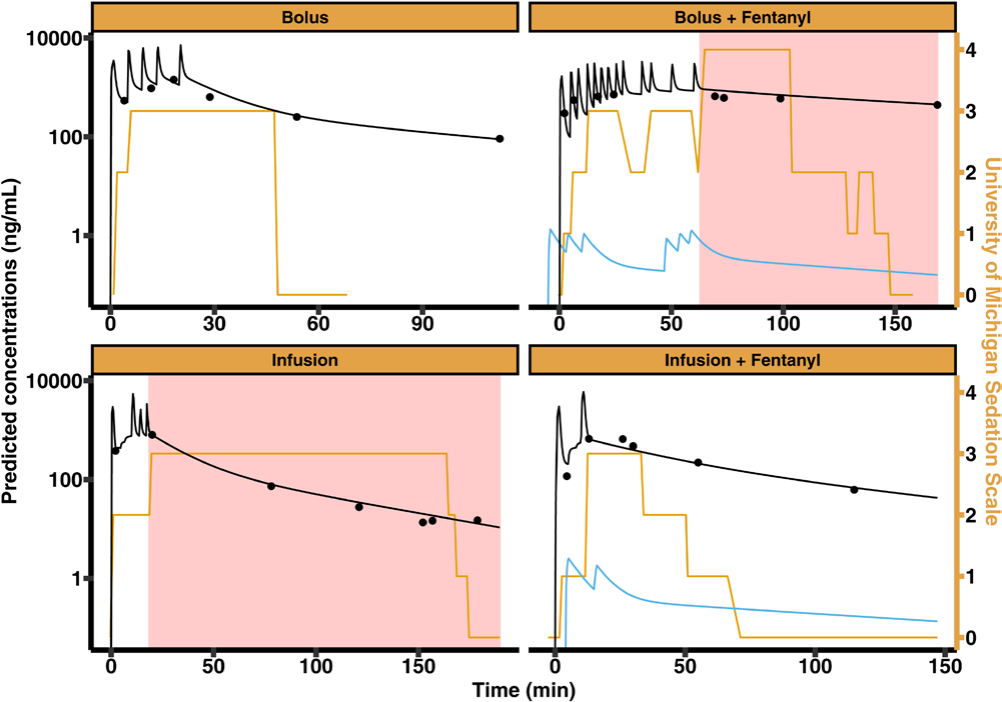
Example of pharmacokinetic pharmacodynamic data collected in this study in representative patients from the studied dosing regimen arms. Measured pharmacokinetic samples are shown with *solid circles*. Observed University of Michigan Sedation Scale scores are depicted with a *solid orange line*. The *pink shaded area* denotes observations that were obtained after rescue medication was used. For context, the remimazolam and fentanyl dosing regimens were visualized using the population pharmacokinetic models developed in this study (*black solid line*) and Ginsberg *et al.*^12^ (*blue solid line*), respectively.

Baseline patient and group characteristics of the 31 patients in this analysis are shown in table 3. The raw pharmacokinetic pharmacodynamic data collected in this study for a representative patient from each group are shown in figure 1, and all individual data are displayed in Supplemental Digital Content 1 figure 1 (https://links.lww.com/ALN/E8). As seen in table 3, rescue medication was used in 29% (9 of 31) of patients and was higher in the two infusion groups (6 of 15; 40%) compared to the two bolus groups (3 of 16; 19%). At the same time, rescue medication was less frequently used in the two groups receiving fentanyl coadministration (5 of 21; 24%) compared to administration of only remimazolam (4 of 10; 40%).

### Safety

Of the 31 patients enrolled, 19 patients experienced one or more adverse events. There were 34 nonserious adverse events and 1 serious adverse event (influenza not related to the investigational medicinal product) reported. A total of 23 adverse events were classified as mild, 12 as moderate, and none as severe by the investigators. A total of 22 adverse events were assessed as related to the investigational medicinal product by the investigators. All adverse events had a resolved outcome. Transient hypotension (systolic or diastolic blood pressure decrease 30% or greater from baseline) was the most frequent adverse event reported, with 20 total incidents of hypotension reported (57.1%; 16 incidents reported as mild and 4 reported as moderate). All but three of these cases were rated as being related to the investigational medicinal product, and all had a resolved outcome. The majority of hypotension adverse events were diastolic hypotension, with 14 cases (70% of reported hypotension cases) reported (11 rated as mild and 3 rated as moderate; 13 related and 1 not related to the investigational medicinal product), and all had a resolved outcome.

### Population Pharmacokinetic Model

A total of 171 blood samples were taken from 31 patients. Of those, 170 remimazolam and 160 CNS7054 concentrations were above the lower limit of quantitation and were included in the analysis. All concentration measurements that were below the lower limit of quantitation were from blood samples taken less than 5 min after the administration of the first remimazolam bolus dose and were ignored in the model development.

A three-compartment model best described the remimazolam pharmacokinetics (ΔOFV against two-compartment model, –13.1; *P* = 0.011 for 4 additional degrees of freedom; two fixed effects and two between-subject variability terms). The disposition kinetics of CNS7054 were best described by a one-compartment model with linear elimination from the central compartment (CL_m_). To attenuate overprediction of CNS7054 concentrations at the early timepoints, a series of five transit compartments were added to the model (ΔOFV, –124; *P* < 0.001). Between-subject variability in the pharmacokinetics was described by implementing a log-normal distribution for remimazolam elimination clearance (CL), the volume of the second peripheral remimazolam compartment (V2), the volume of distribution for CNS7054 (Vm), and CNS7054 CL (CL_m_). Between-subject variability for the other pharmacokinetic parameters in the model was fixed to 10%. None of the covariates were correlated to the *post hoc* pharmacokinetic parameter estimates. Likelihood ratio tests for the effects of age (ΔOFV, –2.21; *P* = 0.13), sex (ΔOFV, –2.38; *P* = 0.12), and fentanyl coadministration (ΔOFV, –0.40; *P* = 0.52) on remimazolam CL were not significant.

**Fig. 2.**
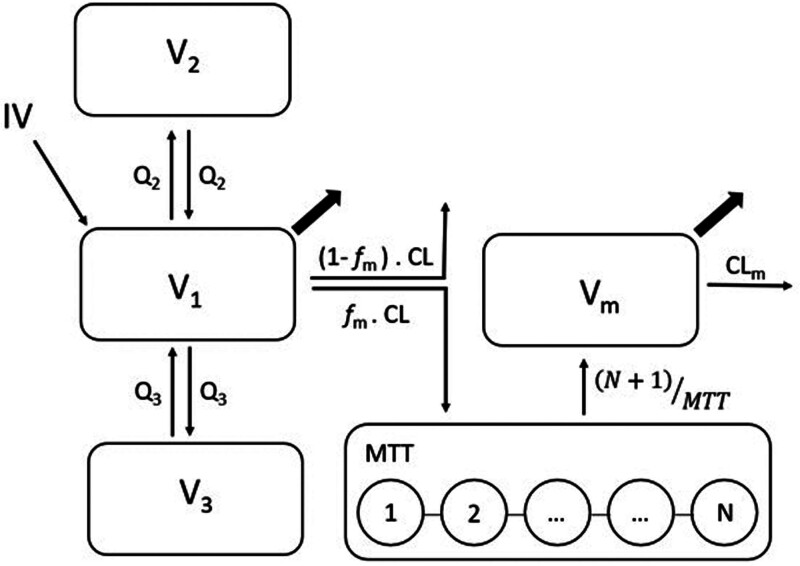
Structure of final joint population pharmacokinetic model for remimazolam and CNS7054. Remimazolam pharmacokinetics are described by a three-compartment model with volumes of distribution V1, V2, and V3 and elimination and intercompartmental clearance CL, Q2, and Q3. CNS7054 pharmacokinetics are described by a one-compartment model with volume of distribution Vm and linear elimination clearance CLm. The formation of CNS7054 is described by a series of transit compartments with N compartments and mean transit time MTT. In this model, we assumed that 100% of remimazolam is metabolized to CNS7054 (*f*_m_ = 1.00). Plasma samples were assumed to be taken from the central compartments for remimazolam (V1) and CNS7054 (Vm) as indicated by the *arrows*.

The structure of the final pharmacokinetic model is shown in figure 2. Parameter estimates and associated 95% CIs are shown in table 4. Negative log-likelihood profiles are shown in Supplemental Digital Content figure 2 (https://links.lww.com/ALN/E8). All parameters were estimated with good precision. A prediction variance–corrected visual predictive check for the final model and goodness-of-fit plots are shown in Supplemental Digital Content figures 3 and 4 (https://links.lww.com/ALN/E8) and demonstrate that the final model describes the observed data well.

**Table 4.** Parameter Estimates for the Population Pharmacokinetic Model of Remimazolam and CNS7054

Parameter Name	Parameter	Estimates	LCB (95% CI)	UCB (95% CI)	IIV	LCB (95% CI)	UCB (95% CI)	Shrinkage (%)
Remimazolam								
Volume of central compartment, l · 70 kg^-1^	V1	1.25	0.77	2.10				
Volume of fast peripheral compartment, l · 70 kg^-1^	V2	10.8	7.45	18.1	92.4	64.3	137	10
Volume of slow peripheral compartment, l · 70 kg^-1^	V3	12.3	8.10	19.3				
Elimination clearance, l · min^-1^ · 70 kg^-1^	CL	0.70	0.52	0.94	63.1	45.8	91.0	3.0
Fast distributional clearance, l · min^-1^ · 70 kg^-1^	Q2	1.68	1.00	2.74				
Slow distributional clearance, l · min^-1^ · 70 kg^-1^	Q3	0.23	0.15	0.33				
Proportional error, %	Prop_RMZ_	41.1	35.2	49.0				
CNS7054								
Volume of central compartment, l · 70 kg^-1^	Vm	12.2	10.2	15.7	49.5	37.5	72.0	5.0
Elimination clearance, l · h^-1^ · 70 kg^-1^*	CLm	4.07	2.52	5.88	64.6	43.6	101	20
Mean transit time, min	MTT	3.97	3.85	5.09				
Proportional error, %	Prop_CNS7054_	17.5	14.8	21.1				
Additive error, ng · ml^-1^	Add_CNS7054_	22.9	14.4	37.2				

The interindividual variability (IIV) was expressed as a coefficient of variation (CV%) calculated as the square root of the variance estimated by NONMEM. The proportional and additive error were expressed as SD. The lower confidence bound (LCB) and upper confidence bound (UCB) of the 95% CI of the parameters were derived using log-likelihood profiling.

* This parameter was estimated in l · min^-1^ · 70 kg^-1^ in the model and is shown in these units in the log-likelihood profiles.

### Pharmacodynamic Model for UMSS

UMSS observations were available from 31 patients and were predominantly from the group receiving fentanyl coadministration (1,394 *vs*. 526 observations). Due to the use of rescue medication, 295 UMSS scores were not used for modeling (235 and 60, respectively). An initial proportional odds logistic regression model not accounting for a potential pharmacodynamic interaction between fentanyl and remimazolam failed to describe the observed UMSS, most notably in the bolus group at predicted effect-site concentrations less than 500 ng · ml^–1^. Accounting for the remimazolam–fentanyl interaction using the simplified Minto model resulted in a decrease in objective function value greater than 20,000 points. When removing the two patients who had a UMSS score greater than 0 after fentanyl dosing but before remimazolam administration, the objective function value for the simplified Minto model remained lower compared to the initial (absence of fentanyl effect) proportional odds logistic regression model (ΔOFV, –152; *P* < 0.001).

A model assuming tolerance development, driven by competitive inhibition by CNS7054, as shown in equations 6 and 7, resulted in the same objective function value (ΔOFV, 0). In this model, the estimated CNS7054 concentration producing half-maximum inhibition (IC50_CNS7054_) was greater than 10-fold higher than the highest observed CNS7054 concentration in the dataset (6,865 ng · ml^-1^). Consequently, the competitive inhibition of CNS7054 was not retained in the final model.

None of the covariates were correlated to the *post hoc* pharmacodynamic parameters. Likelihood ratio tests for the effects of age (ΔOFV, –0.54; *P* = 0.46), sex (ΔOFV, –1.17; *P* = 0.28), fentanyl coadministration (ΔOFV, –2.37; *P* = 0.12), and study procedure (ΔOFV, –0.89; *P* = 0.83 for 3 additional degrees of freedom) on EC50_RMZ_ were not significant.

Parameter estimates for the final UMSS model are shown in table 5 alongside the 95% CI. Log-likelihood profiles are shown in Supplemental Digital Content figure 5 (https://links.lww.com/ALN/E8). All parameters except ΔEC50_FENT,3_ were estimated with good precision as judged by the 95% CIs. For ΔEC50_FENT,3_, the 95% CI was right-skewed (2.59; 41.7 ng · ml^–1^), indicating that it was difficult to precisely estimate the predicted fentanyl effect-site concentration that resulted in a 50% probability of a UMSS score of 3.

**Table 5.** Parameter Estimates for the Pharmacokinetic Pharmacodynamic Model of the University of Michigan Sedation Scale (UMSS)

Parameter Name	Parameter	Estimate	LCB (95% CI)	UCB (95% CI)	IIV	LCB (95% CI)	UCB (95% CI)	Shrinkage (%)
Remimazolam effect-site rate constant	ke0_RMZ_	0.230	0.20	0.27				
Hill coefficient	γ	3.56	3.18	3.96				
EC50 – remimazolam – UMSS 1, ng/ml	EC50_RMZ,1_	206	151	281	68.2	55.9	120.1	32
ΔEC50 – remimazolam – UMSS 2 *vs*. 1, ng/ml	ΔEC50_RMZ,2_	224	162	309				
ΔEC50 – remimazolam – UMSS 3 *vs*. 2, ng/ml	ΔEC50_RMZ,3_	347	249	488				
								
Fentanyl effect-site rate constant	ke0_FENT_	0.125	0.08	0.20				
EC50 – fentanyl – UMSS 1, ng/ml	EC50_FENT,1_	0.560	0.49	0.73				
ΔEC50 – fentanyl – UMSS 2 *vs*. 1, ng/ml	ΔEC50_FENT,2_	1.60	1.09	2.60				
ΔEC50 – fentanyl – UMSS 3 *vs*. 2, ng/ml	ΔEC50_FENT,3_	4.14	2.59	41.7				

The interindividual variability (IIV) was expressed as a coefficient of variation (CV%). The lower confidence bound (LCB) and upper confidence bound (UCB) of the 95% CI of the parameters were derived using log-likelihood profiling.

UMSS, University of Michigan Sedation Scale.

A prediction variance–corrected visual predictive check for the final model is shown in Supplemental Digital Content figure 6 (https://links.lww.com/ALN/E8) and demonstrates that the final model describes the observed UMSS well. All model code is shown in Supplemental Digital Content 1 (https://links.lww.com/ALN/E8).

**Fig. 3.**
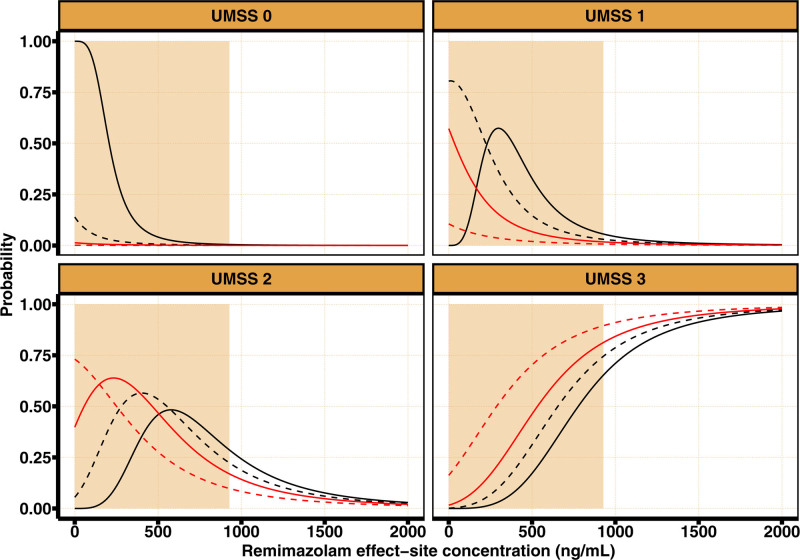
Steady state pharmacodynamic interaction between fentanyl and remimazolam. The predicted probability for University of Michigan Sedation Scale (UMSS) = k with k ∈ {0, 1, 2, 3} for different combinations of remimazolam–fentanyl according to our final pharmacodynamic model. The remimazolam effect-site concentration driving the pharmacodynamic effect is depicted on the *x*-axis. The background fentanyl regimens, expressed as predicted effect-site concentrations, are depicted by different line types: no fentanyl coadministration (*solid black line*), 1 ng · ml^–1^ (*dashed black line*), 2 ng · ml^–1^ (*solid red line*), and 4 ng · ml^–1^ (*dashed red line*). The range of predicted remimazolam effect-site concentrations from this study are denoted by the *orange shaded area*.

Figure 3 illustrates the exposure response relationship derived from our final population pharmacodynamic model. With increasing remimazolam effect-site concentrations, the probability of observing a UMSS score of 3 or greater increases from 1.7% at 250 ng · ml^–1^ to 17% at 500 ng · ml^–1^ and 71% at 1,000 ng · ml^–1^. The EC50 of remimazolam for UMSS score of 3 or greater was 777 ng · ml^–1^ in the absence of fentanyl, and decreased to 655, 533, and 287 ng/ml for concomitant fentanyl steady-state concentrations of 1, 2, or 4 ng · ml^–1^, respectively. At a remimazolam effect-site concentration of 1,000 ng · ml^–1^, fentanyl coadministration increases the probability of observing a UMSS score of 3 or greater from 71 to 79%, 84%, and 91% for predicted fentanyl effect-site concentrations of 1, 2, and 4 ng · ml^–1^, respectively. The effect of fentanyl coadministration decreases with increasing remimazolam effect-site concentrations to about 5% at a remimazolam effect-site concentration of 1,500 ng · ml^–1^ (from 91% without fentanyl to 97% with 4 ng · ml^–1^ fentanyl).

### Performance of Studied Dosing Regimen

As a benchmark to compare modifications, we first simulated the revised remimazolam and fentanyl dosing regimen that was evaluated in our clinical trial (table 2). These simulations showed that for the revised dosing regimen, the percentage of patients not achieving a UMSS score of 3 or greater at the end of the induction (defined as ending 15 min after the start of the first remimazolam bolus) ranges from 9.2 to 22.0% (table 6). These simulations also showed that fentanyl coadministration (1) will result in a 18% (626 *vs*. 762 ng · ml^–1^) and 12% (1,029 *vs*. 1,166 ng · ml^–1^) lower remimazolam exposure for the bolus and bolus plus infusion regimen, (2) will not result in a significant decrease (greater than 0.5%) in the percentage of patients not achieving a UMSS score of 3 or greater, and (3) does not appear to have an effect on the number of top-ups given during the induction and maintenance phase.

**Table 6.** Comparison of the Simulated Outcomes for the Revised Dosing Regimen and the Model-based Optimized Dosing Regimen for Target UMSS Scores 2 to 3

		Bolus Regimen (µg · kg^-1^)	Infusion Rate (µg · kg · min^-1^)	C_p_ (ng · ml^-1^)	UMSS < 3 (%)	Dose Level during Induction (%)				No. of Bolus Doses after Induction (%)			
						1	2	3	>3	0	1	2	>2
Bolus	Without fentanyl	150 q3min*	—	762	21.0	5.2	22.2	31.1	41.6	3.8	29.6	30.3	36.3
		(max. 7.0 mg)											
		200 q3min†	—	787	12.0	10.2	32.7	31.0	26.1	6.9	39.5	30.8	22.8
	With fentanyl	125 q3min*	—	626	22.0	4.2	19.5	31.3	44.9	2.5	25.7	30.8	41.0
		(max. 5.0 mg)											
		200 q3min†	—	713	9.5	12.8	36.7	30.7	19.8	7.7	42.0	30.4	19.9
Bolus	Without fentanyl	150 q3min*	7.5 – 15 – 20	1,166	9.2	15.3	33.5	27.0	24.2	34.3	33.9	17.0	14.8
+ infusion		(max. 7.0 mg)	(max. 1 mg · min^-1^)										
		200 q3min†	10 – 20 – 40 – 80	1,434	3.3	26.3	37.9	24.6	11.3	61.0	29.4	7.6	2.0
	With fentanyl	125 q3min*	7.5 – 15 – 20	1,029	9.8	14.3	33.4	27.9	24.4	30.5	33.0	19.7	16.8
		(max. 5.0 mg)	(max. 1 mg · min^-1^)										
		200 q3min†	10 – 20 – 40 – 80	1,326	2.8	30.4	39.4	22.2	8.0	60.3	30.1	7.6	2.0

Remimazolam was titrated to effect with top-up boluses allowed at 3-min intervals (q3min). The fentanyl dosing regimen was identical between both dosing regimens. For more details, the reader is referred to the “Results” section. C_p_ is the predicted median remimazolam plasma concentration at the end of the induction phase (*i.e.*, 15 min after the first bolus dose of remimazolam is given). UMMS < 3 denotes the predicted percentage of patients not achieving a UMMS score ≥ 3 at the end of the induction phase. “Dose level during induction” denotes the dose level a virtual patient is titrated to during the induction phase to achieve a UMSS score ≥ 3. Virtual patients in dose level 1 were not escalated to a higher dosing regimen. For the “bolus” regimen, this denotes the number of additional bolus doses given. For the “bolus + infusion” regimen, this denotes the number of increments in the per-kilogram infusion rate, accompanied by an additional bolus dose

* Revised dosing regimen.

† Model-based optimized dosing regimen.

UMSS, University of Michigan Sedation Scale.

### Model-based Dosing Regimen Optimization

From the simulations using the UMSS pharmacodynamic model, we found that the time to peak effect was 2.0 min (95% prediction interval, 1.6 to 3.9 min) for remimazolam and 7.0 min for fentanyl. Nevertheless, for practical purposes, when optimizing the dosing regimen, we adhered to the interdose interval for remimazolam top-up bolus from the original dosing regimen (q3min). Fentanyl bolus doses, however, were administered 4 min before the first remimazolam bolus.

The result of this model-based optimization of the dosing regimen is shown in table 6. In the group without fentanyl coadministration, the optimized dosing regimen was simulated to result in a 3.2% (787 *vs*. 762 ng · ml^–1^) and 23% (1,434 *vs*. 1,166 ng · ml^–1^) higher remimazolam exposure for the bolus and bolus plus infusion regimen, respectively. Consequently, the percentage of patients not reaching a UMSS score of 3 or greater after the induction was simulated to decrease by 9.0% (12% *vs*. 21%) and 5.9% (3.3% *vs*. 9.2%). When fentanyl was coadministered according to the optimized dosing regimen, the remimazolam exposure was simulated to decrease by 9.4% (713 *vs*. 787 ng · ml^–1^) for the bolus regimen and by 8.1% (1,326 *vs*. 1,434 ng · ml^–1^) for the bolus plus infusion regimen. At the same time, fentanyl coadministration was simulated to result in a slight decrease in the percentage of patients not achieving a UMSS score of 3 or greater at the end of the induction (9.5% *vs*. 12.0% and 2.8% *vs*. 3.3% for the bolus and infusion scenarios, respectively). Overall, the percentage of patients achieving a UMSS score of 3 or greater was simulated to be high for the optimized dosing regimen and ranged from 88% (for the bolus without fentanyl) to 97% (for the bolus plus infusion regimen with coadministration of fentanyl).

It is noteworthy to appreciate that due to the titration-to-effect element in our simulations, we were also able to evaluate practical consequences of the proposed dosing regimen. On one hand, the optimized bolus dosing regimen reduced the number of top-ups in the induction and maintenance phase due to the higher per-kilogram bolus dose. On the other hand, the optimized infusion regimen was simulated to result in faster titration to effect with more cases requiring no or one infusion rate escalation (26.3% and 37.9% of cases requiring no or one escalation, respectively) compared to the original dosing regimen (15.3% and 33.5% of cases requiring no or one escalation, respectively). Finally, for the infusion with fentanyl coadministration regimen, the number of cases not requiring a top-up in the maintenance phase was simulated to increase 2.0-fold (60.3% *vs*. 30.5%) compared to the original dosing regimen, resulting in a highly practical regimen in which more than 90% of patients require no or one top-up (60.3% and 30.1% of cases requiring 0 or 1 top-up) in the maintenance phase (15 to 30 min after the first remimazolam bolus dose).

**Fig. 4.**
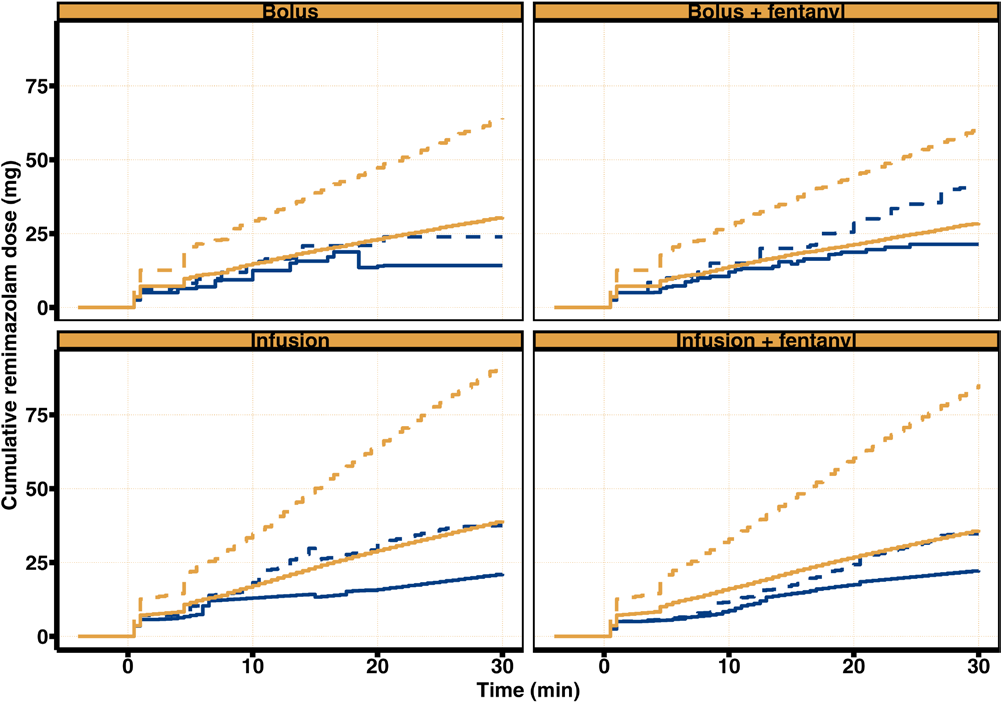
Predicted cumulative remimazolam dose *versus* time for the optimized dosing regimen *versus* the observed cumulative remimazolam dose from this study. *Solid blue and orange lines* represent the median observed cumulative remimazolam dose (milligrams) *versus* time from this study and the predicted cumulative dose according to the optimized dosing regimen, respectively. *Dashed lines* represent the 90% percentile of the observed and predicted cumulative remimazolam doses.

It was observed that a greater proportion of children for whom the dose was capped required rescue sedation. Therefore, during the optimization of the dosing regimen, the decision was made to remove the dose cap that was part of the original and revised dosing protocol as described in tables 1 and 2. To evaluate the effect of removing the dose cap on the expected remimazolam dosing, we calculated, using simulations, the cumulative remimazolam doses that were administered in our virtual population for the optimized dosing regimens and compared this against the cumulative remimazolam doses administered in our clinical study. Figure 4 and table 7 show a comparison between the simulated cumulative doses according to the optimized dosing regimen and the observed cumulative doses for our study. The median cumulative remimazolam dose in our study ranged from 5.5 mg after 5 min in the infusion plus fentanyl group to 22.3 mg after 30 min in the infusion plus fentanyl group. The median simulated cumulative remimazolam dose for the optimized dosing regimen ranges from 9.6 mg after 5 min in the bolus plus fentanyl group to 39.3 mg after 30 min in the infusion group. The differences in cumulative remimazolam exposure between both dosing regimens are more pronounced when comparing the 90% prediction interval for the optimized dosing regimen *versus* the 90th percentile of observed cumulative doses from this study. For example, for the infusion plus fentanyl group, our simulations showed that in 10% of cases, after 30 min, the cumulative remimazolam exposure is expected to exceed 85.3 mg, whereas in this study, the 90th percentile of the observed cumulative exposure in this group was 34.7 mg.

**Table 7.** Predicted Cumulative Remimazolam Dose (Milligrams) for the Optimized Dosing Regimen and the Dosing Regimen Evaluated in this Study

	Bolus Group	Bolus + Fentanyl Group	Infusion Group	Infusion + Fentanyl Group
5 min				
Optimized dosing	10.3 [20.6]	9.6 [19.3]	11.4 [21.9]	10.7 [20.8]
This study	6.4 [8.2]	7.0 [10.0]	7.1 [10.2]	5.5 [5.9]
10 min				
Optimized dosing	14.9 [29.3]	13.8 [27.8]	17.2 [34.6]	16.2 [33.0]
This study	12.5 [14.3]	12.0 [15.0]	13.0 [18.2]	8.7 [11.6]
20 min				
Optimized dosing	23.1 [47.3]	21.4 [44.4]	29.1 [64.9]	26.9 [60.3]
This study	14.0 [21.3]	18.7 [28.5]	15.8 [31.6]	17.5 [24.3]
30 min				
Optimized dosing	30.6 [64.1]	28.6 [60.8]	39.3 [92.4]	36.0 [85.3]
This study	14.2 [23.9]	21.4 [40.5]	21.2 [37.5]	22.3 [34.7]

Numbers represent the median observed or predicted cumulative remimazolam dose (milligrams) and, between brackets, the 90% percentile of the observed or predicted cumulative remimazolam dose (milligrams).

## Discussion

Our pharmacokinetic–pharmacodynamic analysis showed that the original and revised dosing regimens used in our study were unlikely to be effective in achieving the targeted depth of sedation (UMSS scores of 3 or greater) in pediatric patients 6 yr or older. After careful evaluation of the pharmacokinetic and pharmacodynamic data, an optimized dosing regimen was presented: higher per-kilogram bolus doses and infusion rates, an extended titration scheme for continuous infusion dosing, and removal of the maximum allowable dose (“dosing cap”).

The original remimazolam dosing regimen for pediatric patients in this clinical trial was selected to match the remimazolam exposure in adults receiving the approved dosing regimen for procedural sedation. To reduce the influence of body weight on exposure, pediatric patients were dosed on a per-kilogram basis with a maximum allowable absolute dose not exceeding the approved adult dose. Gao *et al.*^5^ estimated a remimazolam elimination clearance of 0.73 l · min^–1^ · 70 kg^–1^ and suggested that the pharmacokinetics of remimazolam are likely not very different in the pediatric population aged 2 to 6 yr and adults, after correcting for differences in body size. In this study, we estimated a similar remimazolam elimination clearance of 0.70 l · min^–1^ · 70 kg^–1^ (95% CI, 0.51 to 0.94 l · min^–1^ · 70 kg^–1^) in pediatric patients aged 6 yr and older. Nonetheless, Zhou *et al.*^17^ have previously reported a remimazolam elimination clearance of 1.18 l · min^–1^ · 70 kg^–1^ in adult patients undergoing procedural sedation and 1.03 l · min^–1^ · 70 kg^–1^ in adult patients under general anesthesia. This suggests that remimazolam elimination clearance is likely lower (32 to 41%) in pediatric patients compared to adult patients. The reasons for this are currently unclear. In contrast to patients included in the study by Gao *et al.*^5^ (mean body weight of 18 kg), pediatric patients in this clinical trial (mean weight of 50.6 kg with a range from 20.5 to 106 kg) had a body weight similar to the adult population studied by Zhou *et al.*^17^ (median weight, 64.2 kg for procedural sedation patients; mean weight, 76 kg for general anesthesia patients). Based on the pharmacokinetics, after correcting for body size, this would imply that the optimal dose for procedural sedation in the pediatric population is lower than in adults, which is the opposite of what we observed in this study.

Hence, the differences in pharmacokinetic profile between pediatric patients and adults are unable to explain why the success rate in adults was considerably higher even with a lower number of top-ups before the start of the procedure.^18,19^ Other contributing factors for this difference that we observed could be attributed to the difference in the pharmacodynamic endpoints in adults and children. In adults, the 6-point Modified Observer’s Assessment of Alertness and Sedation (MOAA/S)^20^ scale was used, and a MOAA/S of 3 to 4 (mild to moderate sedation) was targeted in the pivotal procedural sedation efficacy trials (NCT02296892 and NCT02290873). In the current trial in children, the 5-point UMSS scale was used, and a UMSS score of 2 to 3 was targeted. In fact, mild sedation (UMSS, 1 to 2) was within the scope of the current trial but not chosen, indicating that mild sedation was not adequate for most of these procedures. In addition to the difference in pharmacokinetics, the exposure–response relationship may differ between children and adults. Establishing the similarity of the exposure–response relationship based on this study is difficult due to the investigators’ need to target deeper levels of sedation in children compared to the level of sedation required in the adult clinical trials. In fact, in this trial, when patients were rescued for inadequate sedation, the investigators ended up continuing the sedation at a UMSS score of 4 (unarousable to deep stimuli).

The pharmacodynamic interaction between fentanyl and remimazolam on UMSS were modeled in this study by an interaction model proposed by Minto *et al.*^13^ to describe additive drug interactions (*i.e.*, assuming β_2,U50_ = β_3,U50_ = β_4,U50_ = 0). The model by Minto *et al.*^13^ allows testing for synergism or infraadditivity by estimating parameters β_2,U50_, β_3,U50_, and β_4,U50_ from the data. An unbiased estimate of the degree of synergism and/or infraadditivity requires that data are available on the drug effects of both drugs administered separately. In this study, such data are not available for fentanyl. Therefore, we refrained from testing for synergism and/or infraadditivity. At the same time, we acknowledge that our estimates for the fentanyl drug effects (EC50_FENT,1_, ΔEC50_FENT,2_, ΔEC50_FENT,3_) are likely confounded and may not extrapolate well to situations where fentanyl is administered in the absence of remimazolam. Using the model by Minto *et al.*,^13^ Zhou *et al.*^21^ have previously shown an infraadditive interaction (β_2,U50_ = –2.20; table 1 in Zhou *et al.*^21^) between remimazolam and fentanyl when using MOAA/S to target particular sedation depths in adults. We hypothesize that the analysis by Zhou *et al.*^21^ suffered from the same confounding as ours, which led to the counterintuitive conclusions of infraadditivity despite previous reports on the synergistic nature of the interaction between benzodiazepines and opioids.^13^

A quantitative comparison between our model and the model by Zhou *et al.*^21^ is difficult because of differences in model structure (Zhou *et al.*^21^ used a discrete time Markov model) and the sedation scales used. One obvious difference between both analyses is that the rate constants for effect-site equilibration from our study for remimazolam and fentanyl are smaller than those reported by Zhou *et al.*^21^ (0.619 min^–1^ *vs*. 0.230 min^–1^ and 0.569 min^–1^ *vs*. 0.125 min^–1^, respectively). As a result, the optimized dosing regimen presented here suggests administering fentanyl 4 min before remimazolam dosing and not 2 min before, as suggested by Zhou *et al.*^17,21^

Vellinga *et al.*^9,22^ have previously shown that CNS7054 is a competitive antagonist to remimazolam. In this study, we were not able to quantify the influence of CNS7054 on the pharmacodynamic effects of remimazolam. Modifications to the UMSS model to account for the CNS7054 – remimazolam interaction failed, mainly due to issues with model stability and parameter estimation. We hypothesize that this is because CNS7054 concentrations in this study (median, 1,539 ng · ml^–1^) were significantly lower than those in the study by Vellinga *et al.*^9,22^ (median, 9,176 ng · ml^–1^). As the apparent CNS7054 elimination clearance for both populations are similar (3 l · h^–1^ · 70 kg^–1^ for Vellinga *et al.*^22^ and 4.07 l · h^–1^ · 70 kg^–1^ for this study, with overlapping 95% CIs), the concentration difference is most likely explained by the higher remimazolam exposure (with target effect-site concentrations up to 2,000 ng · ml^–1^ *vs*. median concentration of 315 ng · ml^–1^ in this study) and longer treatment duration in the study by Vellinga *et al.*^9,22^

During our analysis, we learned that there is no need for per-protocol dose adjustments to account for potential pharmacodynamic drug interactions when designing a dosing regimen for a drug that is titrated to effect. Model simulations show that in the presence of fentanyl, fewer remimazolam bolus doses are given in the induction and maintenance phase, fewer patients are titrated to the highest allowable remimazolam infusion rate, and remimazolam exposure is 9.4% and 8.1% lower in the bolus and bolus plus infusion regimens when fentanyl is coadministered. This intuitive dose reduction is the result of the titration to effect (top-up doses were added to the simulations when sedation was predicted to be below the target level) that is integrated in the simulations and is informed by the strength of the pharmacodynamic interaction in the UMSS model. An advantage of our approach is that it allows for a harmonized dosing regimen, irrespective of fentanyl coadministration, which likely facilitates the implementation of the dosing regimen in a clinical trial and in clinical practice. In other situations where the magnitude of the pharmacodynamic interaction is larger or a smaller narrow therapeutic–toxic margin exists, this approach may not work.

Our study has several limitations. First, the initial clinical trial protocol was developed to achieve mild to moderate sedation depths. Consequently, no UMSS scores of 4 were observed (before the administration of rescue medication), we were not able to model the probability of achieving a UMSS score of 4, and our simulations could not inform on the likelihood of too deep sedation. Second, for the optimized dosing regimen, in which the dose cap was removed, model simulations indicate higher cumulative dosages in some pediatric patients to achieve adequate sedation. New clinical data are necessary to confirm that the optimized dosing regimen is acceptable in terms of safety. Third, no fentanyl concentrations were measured in our study. Instead, we used a pharmacokinetic model for pediatric patients published by Ginsberg *et al.*^12^ to predict the time course of fentanyl concentrations. Potential bias in this model may have confounded the reported parameter estimates and may limit our model’s generalizability to situations in which different fentanyl dosing regimens are used. Therefore, in this study, we refrained from optimizing the fentanyl dosing regimen. Fourth, higher fentanyl doses are required to better characterize the remimazolam–fentanyl interaction at UMSS scores greater than 2 and to attain a more precise estimate of ΔEC50_FENT,3_ in the model. When interpreting our simulations for UMSS scores of 3 or greater, this uncertainty should be taken into account. Fifth, we did not measure the effect of fentanyl on the UMSS in the absence of remimazolam. As a consequence, our analysis did not allow us to reliably test for synergism or infraadditivity in the combined drug effects of remimazolam and fentanyl on UMSS.

In conclusion, we have demonstrated that to effectively use remimazolam for procedural sedation in children 6 to 18 yr old, the dosing regimen that was originally proposed in the U.S. Food and Drug Administration pediatric study plan should be modified to allow for higher remimazolam and fentanyl exposures. The optimized dosing regimen is predicted to translate to better conditions for procedural sedation, but should be further confirmed using clinical data.

### Acknowledgments

The authors thank the trial investigators: R. J. Ramamurthi, M.D. (Stanford University, Stanford, California), Kumar Belani, M.D. (University of Minnesota, Minneapolis, Minnesota), Peter Davis, M.D. (University of Pittsburgh, Pittsburgh, Pennsylvania), and Chyongiy (Joyce) Liu, D.O., M.S., M.B.A. (Baylor College of Medicine, Houston, Texas). The authors also thank Iris Kleine and Ladislav Raschka, Ph.D. (both PAION UK Ltd., London, United Kingdom), and Daniel Harris, B.S.N., and Michael Conover, M.S., M.B.A. (both Eagle Pharmaceuticals, Woodcliff Lake, New Jersey), for their clinical trial operational expertise and guidance.

### Research Support

The clinical trial was sponsored by Acacia Pharma Ltd. (Cambridge, United Kingdom, a wholly owned subsidiary of Eagle Pharmaceuticals, Woodcliff Lake, New Jersey) and PAION UK Ltd. (London, United Kingdom). The modeling and simulation analysis was supported by Acacia Pharma Ltd. (Cambridge, United Kingdom) through a contract research agreement with University Medical Center Groningen, Groningen, The Netherlands.

### Competing Interests

Dr. Struys declares that his research group/department received (during the last 3 yr) research grants and consultancy fees from The Medicines Company (Parsippany, New Jersey), Masimo (Irvine, California), Becton Dickinson (Eysins, Switzerland), Fresenius (Bad Homburg, Germany), Dräger (Lübeck, Germany), Paion (Aachen, Germany), and Medtronic (Dublin, Ireland). He receives royalties on intellectual property from Demed Medical (Temse, Belgium) and Ghent University (Ghent, Belgium). He is an editorial board member and director for the *British Journal of Anaesthesia*. Dr. Colin declares that during the last 3 yr, his research group has been involved in contract research for PAION UK Ltd. (London, United Kingdom) and Acacia Pharma Ltd. (Cambridge, United Kingdom). Dr. Bichajian and Dr. Curt are employees of Eagle Pharmaceuticals (Woodcliff Lake, New Jersey), and Dr. Stöhr is an employee of Paion (Aachen, Germany). Eagle Pharmaceuticals and Paion are the cosponsoring companies of the trial. The other authors declare no competing interests.

### Reproducible Science

Full protocol available at: lbichajian@eagleus.com. Raw data available at: lbichajian@eagleus.com.

## Supplemental Digital Content

Supplemental material, https://links.lww.com/ALN/E8

Supplemental Digital Content Figure 1: Pharmacokinetic pharmacodynamic data collected in this study.

Supplemental Digital Content Figure 2: Negative log-likelihood profiles for the remimazolam–CNS7054 population pharmacokinetic model

Supplemental Digital Content Figure 3: Prediction variance–corrected visual predictive check for the remimazolam–CNS7054 population pharmacokinetic model

Supplemental Digital Content Figure 4: Goodness-of-fit plots for the final population pharmacokinetic model for remimazolam and CNS7054.

Supplemental Digital Content Figure 5: Negative log-likelihood profiles for the UMSS population pharmacodynamic model

Supplemental Digital Content Figure 6: Prediction variance–corrected visual predictive check for the UMSS population pharmacodynamic model

Supplemental Digital Content Table 1: University of Michigan Sedation Scale

Supplemental Digital Content 1: NONMEM code for sequential PKPD model for UMSS in pediatric patients.

## Supplementary Material
