# Supplementary material for: Pharmacokinetics and Pharmacodynamics of Remimazolam for Procedural Sedation in Children and Adolescents
Source: Anesthesiology. 2025 May 12;143(2):368–82. doi: 10.1097/ALN.0000000000005560 (PMC12227205; doi:10.1097/ALN.0000000000005560)
Supplement: Supplementary file 1 [file aln-143-368-s001.pdf]

Supplemental Digital Content 1

Figure 1. Pharmacokinetic pharmacodynamic data collected in this study. Measured PK samples are shown with solid circles, observed UMSS are depicted with a solid orange line. The pink shaded area denotes observations that were obtained after rescue medication was used. For context, the remimazolam and fentanyl dosing regimens were visualized using the post hoc PK parameters estimated with our final population PK model (black solid line) and Ginsberg et al.[11] (blue solid line), respectively.

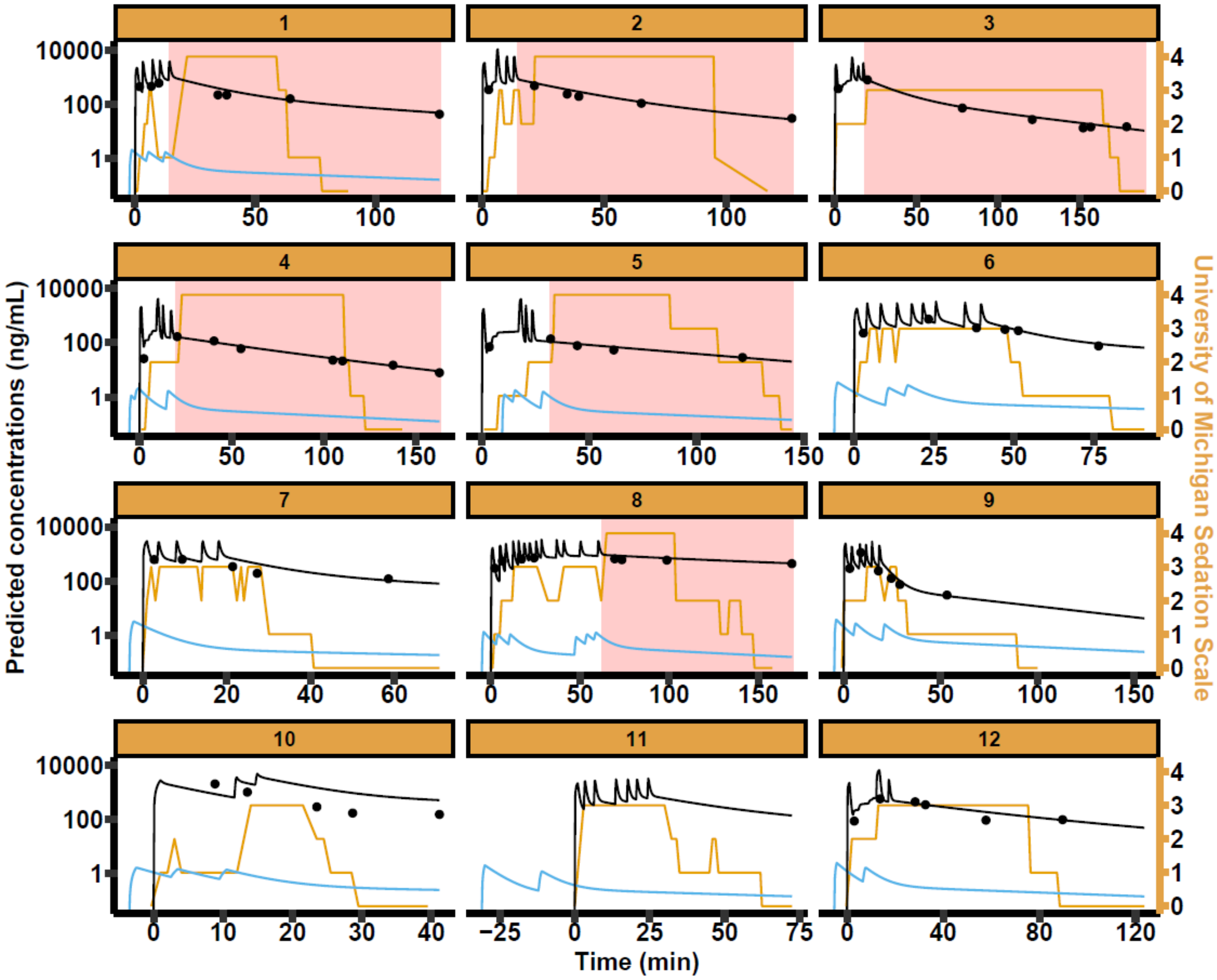

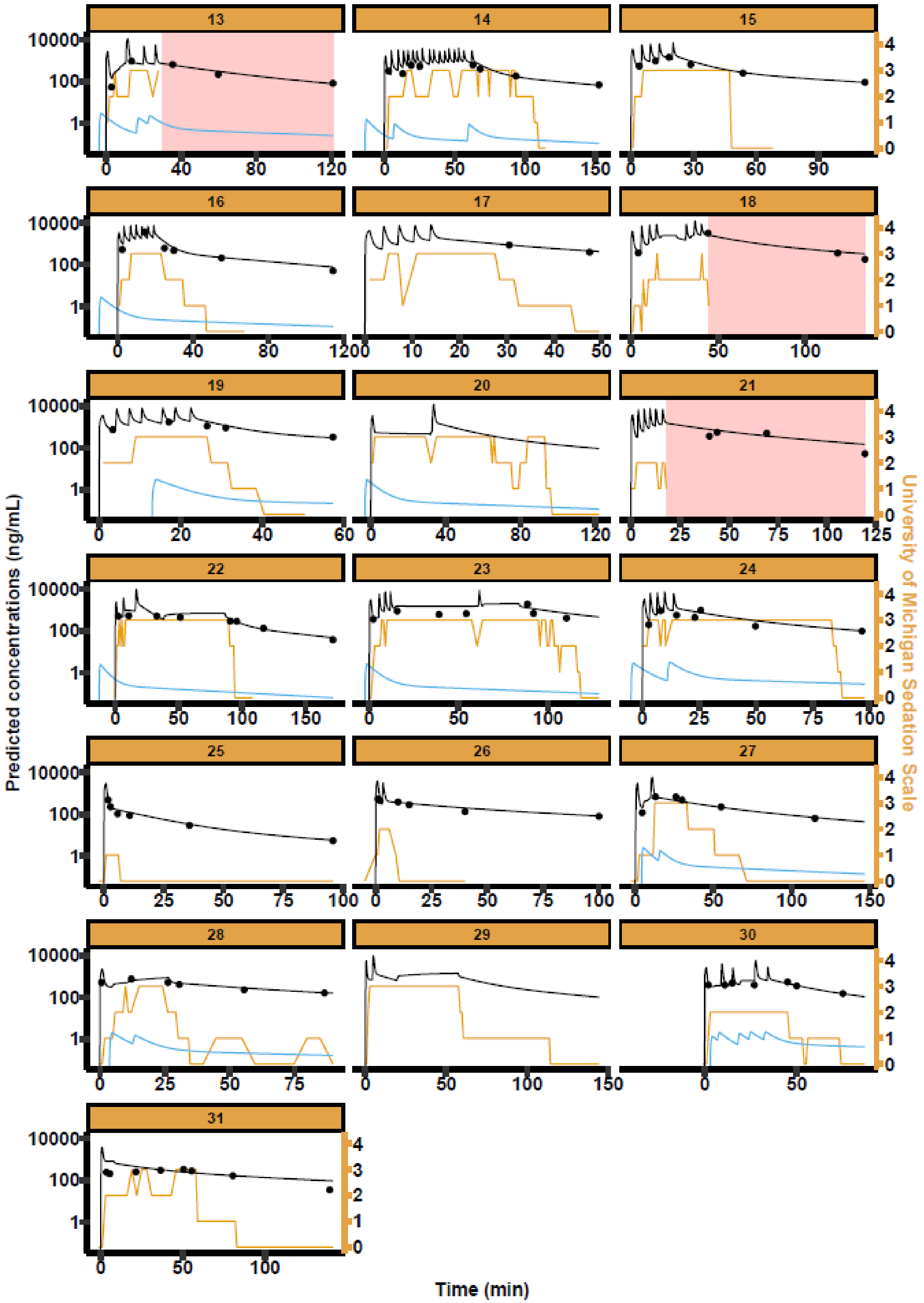
13
14
15
16
17
18
19
20
21
22
23
24
25
26
27
28
29
30
31
Predicted concentrations (ng/mL)
University of Michigan Sedation Scale
Time (min)

Figure 2. Negative log-likelihood profiles – THETAs, OMEGAs and SIGMAs – final population pharmacokinetic model. THETA1 = V1, THETA2 = V2, THETA3 = V3, THETA4 = CL, THETA5 = Q2, THETA6 = Q3, THETA7 = Vm, THETA8=CLm, THETA9 = MTT, THETA10 = Proportional error, THETA12 = Proportional error – metabolite, THETA13 = Additive error – metabolite, OMEGA.2.2. = V2, OMEGA.4.4. = CL, OMEGA.7.7. = Vm, OMEGA.8.8. = CLm

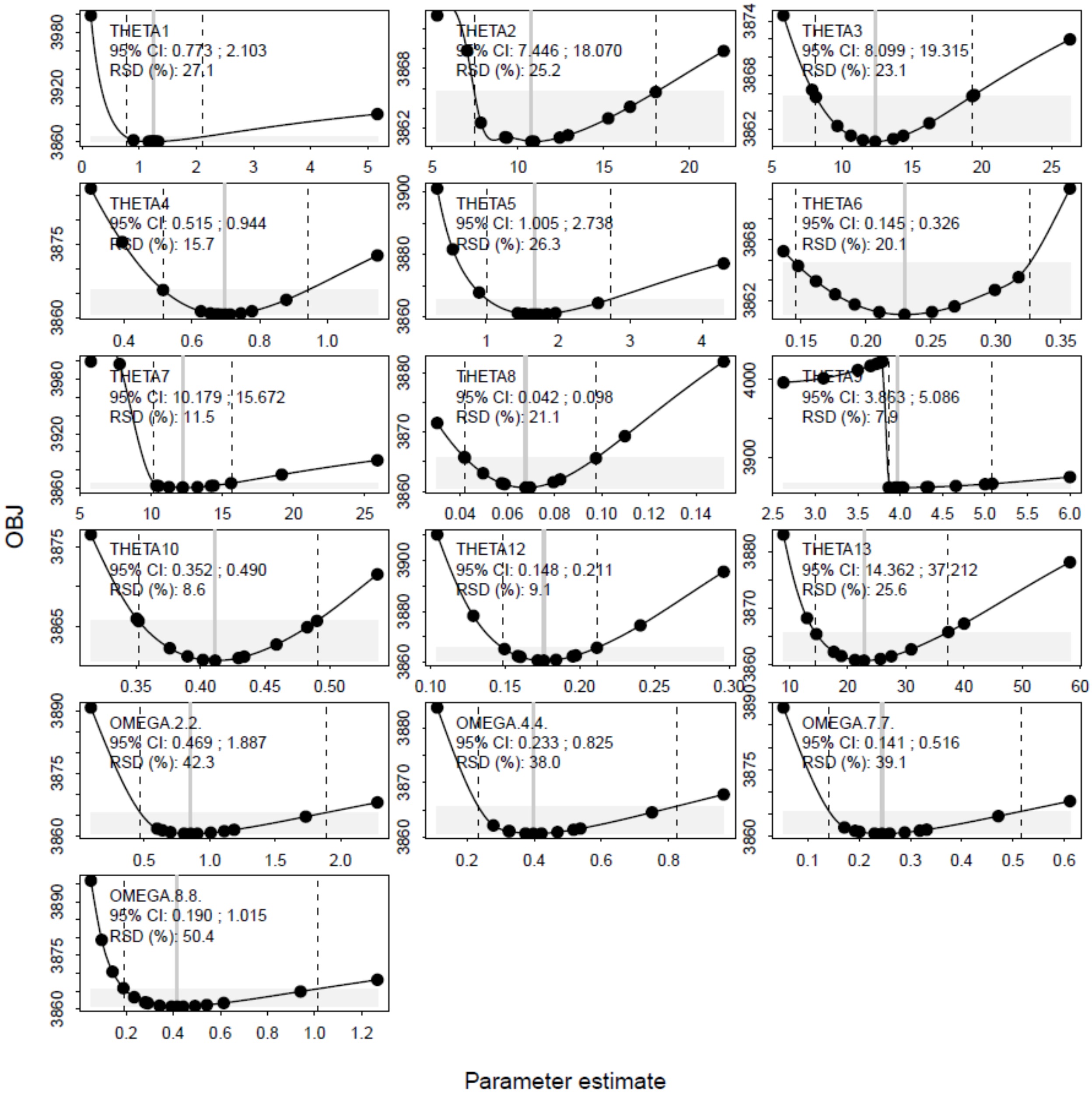

Figure 3. Prediction-variance-corrected Visual Predictive Check (pvcVPC) for the final population pharmacokinetic model. *The dashed blue line represents the median of the prediction-variance-corrected observed concentrations. The solid orange line denotes the median of the prediction-variance-corrected predicted concentrations. Orange shaded areas represent the 95% prediction interval for the simulated median according to the final model. The x-axis denotes the timing of the blood sample relative to the end of the preceding remimazolam dose with positive times for samples taken after a bolus dose and negative times for samples taken before the end of a preceding infusion.*

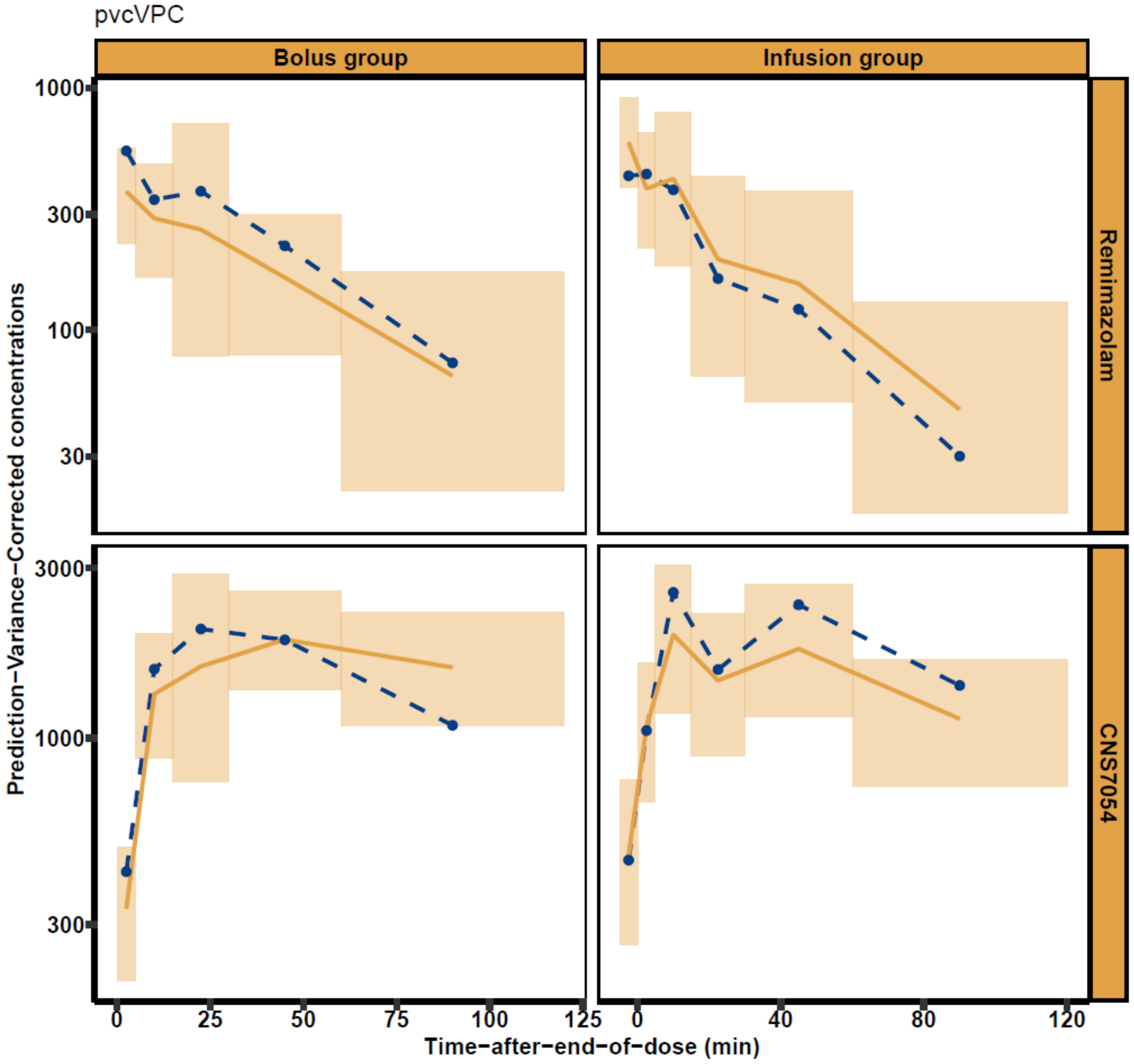

Figure 4. Goodness-of-fit plots for the final population pharmacokinetic model for remimazolam (Panel A) and CNS7054 (Panel B). The top panels depict the population (left) and individual (right) predictions on the x axis against the observed plasma concentrations. The middle panels show the absolute value of the normalized prediction distribution errors (NPDE) against the population predictions (left) and time (right) and the bottom panels show the normalized prediction distribution errors against the population predictions (left) and time (right). The solid red line is a non-parametric smoother (LOESS) to the data.

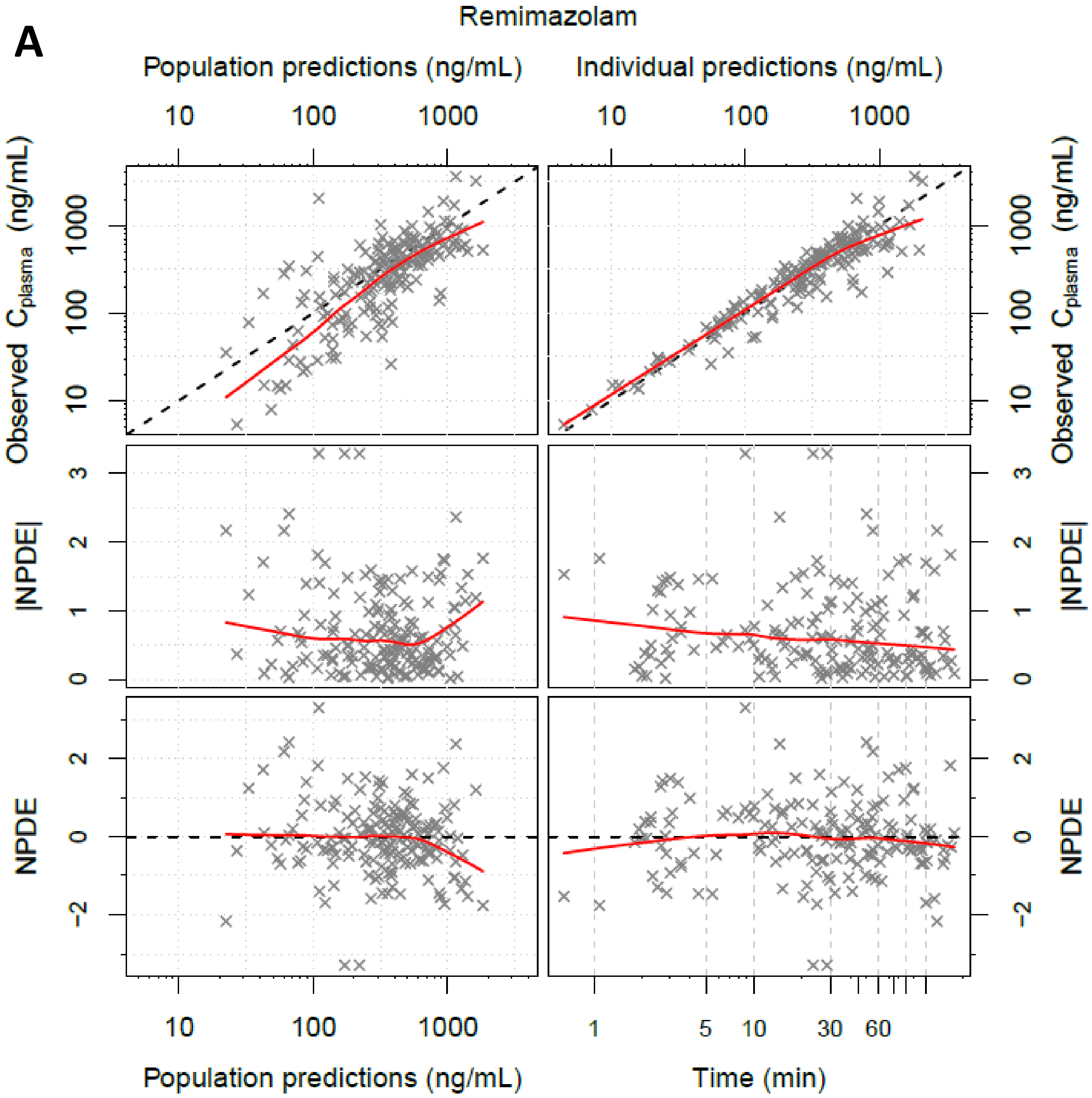

B

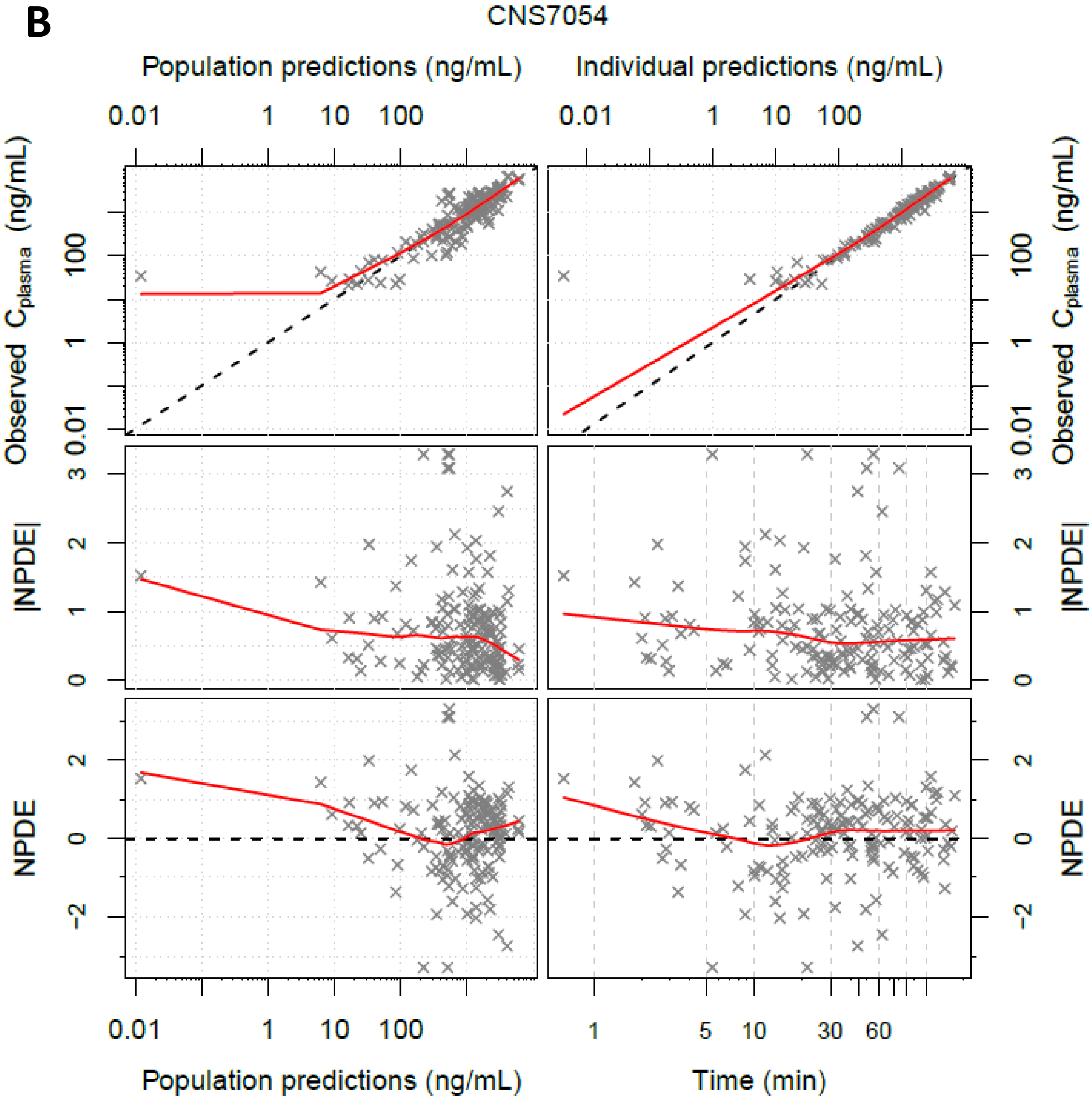
CNS7054
Population predictions (ng/mL)
Individual predictions (ng/mL)
0.01
1
10
100
Observed C_{plasma} (ng/mL)
|NPDE|
NPDE
0
1
2
3
-2
Time (min)
5
30
60

Figure 5. Negative og-likelihood profiles – THETAs, OMEGAs and SIGMAs – final population pharmacodynamic model for UMSS. THETA14 = ke0RMZ, THETA15 = Hill coefficient, THETA16 = EC50 – remimazolam – UMSS 1, THETA17 = ΔEC50 – remimazolam – UMSS 2 vs 1, THETA18 = ΔEC50 – remimazolam – UMSS 3 vs 2, THETA19 = ke0fent, THETA20 = EC50 – fentanyl – UMSS 1, THETA21 = ΔEC50 – fentanyl – UMSS 2 vs 1, THETA22 = ΔEC50 – fentanyl – UMSS 3 vs 2, OMEGA.1.1. = EC50 – remimazolam – UMSS 1

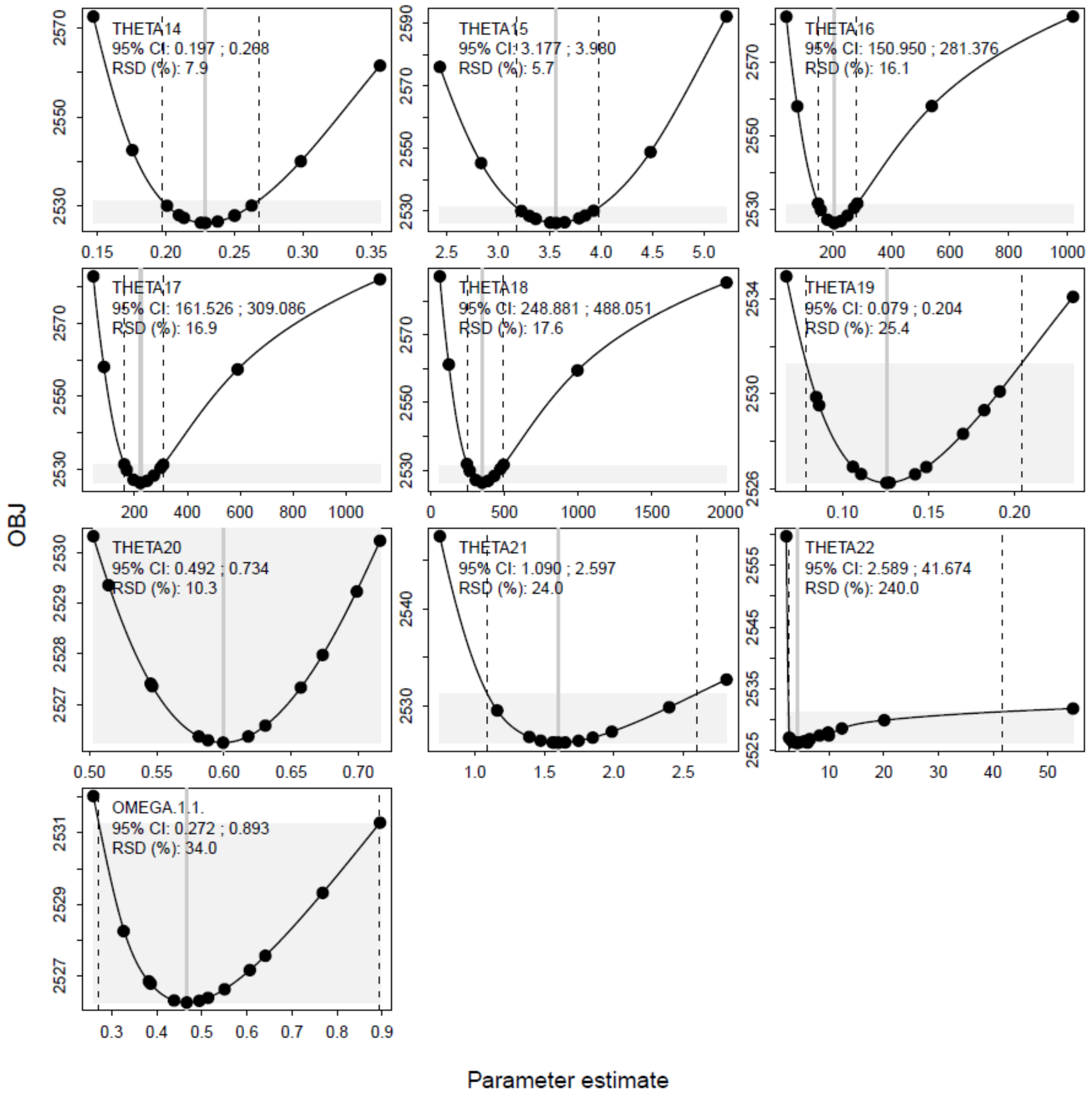

Figure 6. Prediction-variance-corrected Visual Predictive Check (pvcVPC) for the final population pharmacodynamic model for UMSS. *The dashed blue line represents the median of the prediction-variance-corrected observed UMSS. The solid orange line denotes the median of the prediction-variance-corrected predicted UMSS. Orange shaded areas represent the 95% prediction interval for the simulated median according to the final model. The x-axis denotes the predicted remimazolam effect-site concentration (Ce).*

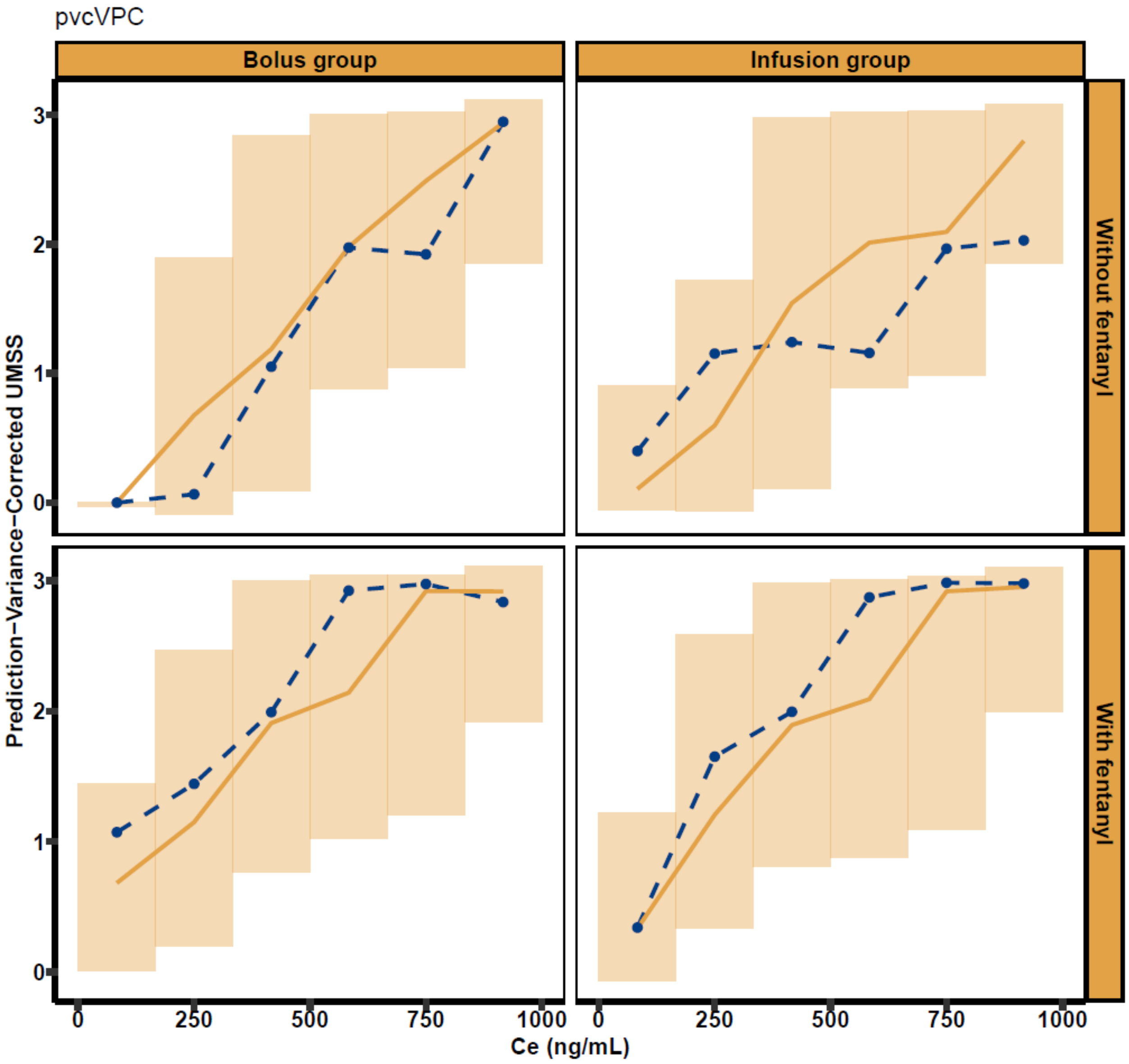

Table 1. University of Michigan Sedation Scale

0	Awake and alert
1	Minimally sedated: tired/sleepy, appropriate response to verbal conversation and/or sound
2	Moderately sedated: somnolent/sleeping, easily aroused with light tactile stimulation or a simple verbal command
3	Deeply sedated: deep sleep, arousable only with significant physical stimulation
4	Unarousable

NONMEM code for final PopPK model in paediatric patients.

```
$SUBROUTINE ADVAN6 TOL=9

$MODEL     COMP(CENTRAL) COMP(PERIPH) COMP(PERIPH2) COMP(MET) COMP(TRANS1)
COMP(TRANS2) COMP(TRANS3) COMP(TRANS4) COMP(TRANS5)

$PK

FSIZE = WEIGHT/70

V1   = EXP(THETA(1)+ETA(1))*FSIZE

V2   = EXP(THETA(2)+ETA(2))*FSIZE

V3   = EXP(THETA(3)+ETA(3))*FSIZE

CL   = EXP(THETA(4)+ETA(4))*FSIZE**0.75

Q2   = EXP(THETA(5)+ETA(5))*FSIZE**0.75

Q3   = EXP(THETA(6)+ETA(6))*FSIZE**0.75

Vm   = EXP(THETA(7)+ETA(7))*FSIZE

CLm  = EXP(THETA(8)+ETA(8))*FSIZE**0.75

N   = 5

MTT = EXP(THETA(9)+ETA(9))

k12 = Q2/V1

k21 = Q2/V2

k10 = CL/V1

k13 = Q3/V1

k31 = Q3/V3

k40 = CLm/Vm

ktr = (N+1)/MTT

PROP = EXP(THETA(10))

ADD  = EXP(THETA(11))

PROP_MET = EXP(THETA(12))

ADD_MET  = EXP(THETA(13))
```

```
$DES

DADT(1)= -k12*A(1) +k21*A(2) -k13*A(1) +k31*A(3) -k10*A(1)

DADT(2)=  k12*A(1) -k21*A(2)

DADT(3)=                    k13*A(1) -k31*A(3)

DADT(4)=  ktr*A(9) - k40*A(4)

DADT(5) = k10*A(1) -ktr*A(5)

DADT(6) =          ktr*A(5) -ktr*A(6)

DADT(7) =                   ktr*A(6) -ktr*A(7)

DADT(8) =                            ktr*A(7) -ktr*A(8)

DADT(9) =                                      ktr*A(8) -ktr*A(9)


$ERROR

PARENT = (A(1)/V1)*1000            ; Conversion from µg.mL-1 to ng.mL-1

MET    = (A(4)/Vm)*1000*439.3/425.3 ; to account for difference in MW

IPRED = PARENT*(2-DVTY) + MET*(DVTY-1)

SD = SQRT(IPRED*IPRED*PROP*PROP+ADD*ADD)*(2-DVTY) +
SQRT(IPRED*IPRED*PROP_MET*PROP_MET+ADD_MET*ADD_MET)*(DVTY-1)

Y     = IPRED + SD*EPS(1)

IRES = DV-IPRED


$THETA

(-1.2, 0.8, 3) ; V1; L

(0.5, 2.5, 4.5)  ; V2; L

(0.5, 2.5, 4.5)  ; V3; L

(-2.2, -0.2, 2) ; CL; L.min-1

(-1.5, 0.5, 2.5); Q2; L.min-1

(-4, -2, 0.5) ; Q3; L.min-1

(-2, 1, 3)  ; Vm; L

(-4, -1, 1.5)    ; CLm; L.min-1
```

```
(-0.5, 1.5, 2.5) ; MTT; min

-0.5      ; Prop.error; ...

0 FIX     ; Add.error; ng/mL-1 ;; SD = 1 ng.mL-1

-0.5      ; Prop.error_met; ...

5         ; Add.error_met; ng/mL-1


$OMEGA

1E-2 FIX ; IIV_V1

0.1     ; IIV_V2

1E-2 FIX ; IIV_V3

0.1     ; IIV_CL

1E-2 FIX ; IIV_Q2

1E-2 FIX ; IIV_Q3

0.1     ; BSV_Vm

0.1     ; BSV_CLm

1E-2 FIX ; BSV_MTT


$SIGMA

1 FIX


$EST METHOD=CONDITIONAL INTERACTION PRINT=1 SIG=3 SIGL=9 MAX=9999 NOABORT

$COV PRINT=E
```

NONMEM code for final PKPD model for UMSS in paediatric patients.

```
$SUBROUTINE ADVAN6 TOL=9


$MODEL     COMP(CENTRAL) COMP(PERIPH) COMP(PERIPH2) COMP(MET) COMP(TRANS1)
COMP(TRANS2) COMP(TRANS3) COMP(TRANS4) COMP(TRANS5) COMP(FENT_C) COMP(FENT_P)
COMP(EFFECT) COMP(EFFECT_FENT)
```

```
$PK
FSIZE = WEIGHT/70

V1   = EXP(THETA(1)+IETA1)*FSIZE
V2   = EXP(THETA(2)+IETA2)*FSIZE
V3   = EXP(THETA(3)+IETA3)*FSIZE
CL   = EXP(THETA(4)+IETA4)*FSIZE**0.75
Q2   = EXP(THETA(5)+IETA5)*FSIZE**0.75
Q3   = EXP(THETA(6)+IETA6)*FSIZE**0.75

Vm   = EXP(THETA(7)+IETA7)*FSIZE
CLm  = EXP(THETA(8)+IETA8)*FSIZE**0.75

N   = 5
MTT = EXP(THETA(9)+IETA9)

k12 = Q2/V1
k21 = Q2/V2
k10 = CL/V1
k13 = Q3/V1
k31 = Q3/V3
k40 = CLm/Vm
ktr = (N+1)/MTT

PROP = EXP(THETA(10))
ADD  = EXP(THETA(11))
PROP_MET = EXP(THETA(12))
ADD_MET  = EXP(THETA(13))
```

```
;-------------------- UMSS Effect compartment ------------------------------------------------
ke0  = EXP(THETA(14))
GAMMA = EXP(THETA(15)+ETA(2))

;-------------------- Fentanyl PK ------------------------------------------------
ke01 = EXP(THETA(19))

CL_FENT = 0.019*WEIGHT
V10 = 0.32*WEIGHT
V11 = 1.49*WEIGHT
Q11 = 0.036*WEIGHT

k1011 = Q11/V10
k1110 = Q11/V11
k100  = CL_FENT/V10

$DES
DADT(1)= -k12*A(1) +k21*A(2) -k13*A(1) +k31*A(3) -k10*A(1)
DADT(2)=  k12*A(1) -k21*A(2)
DADT(3)=                  k13*A(1) -k31*A(3)
DADT(4)=  ktr*A(9) - k40*A(4)
DADT(5) = k10*A(1) -ktr*A(5)
DADT(6) =          ktr*A(5) -ktr*A(6)
DADT(7) =                  ktr*A(6) -ktr*A(7)
DADT(8) =                          ktr*A(7) -ktr*A(8)
DADT(9) =                                  ktr*A(8) -ktr*A(9)
DADT(10)= -k1011*A(10) +k1110*A(11) -k100*A(10)
DADT(11)=  k1011*A(10) -k1110*A(11)
DADT(12)= ke0*((A(1)/V1)-A(12))
```

```
DADT(13)= ke01*((A(10)/V10) - A(13))

$ERROR
PARENT = (A(1)/V1)            ; Conversion from µg.mL-1 to ng.mL-1
MET    = (A(4)/Vm)*439.3/425.3 ; to account for difference in MW
CE     = A(12)

FENT = 0
IF(OPIOID.EQ.1) FENT  = A(10)/V10
CE_FENT = 0
IF(OPIOID.EQ.1) CE_FENT = A(13)          ; ng/mL

TVEC50_1 = EXP(THETA(16))
TVEC50_2 = TVEC50_1 + EXP(THETA(17))
TVEC50_3 = TVEC50_2 + EXP(THETA(18))

EC50_1 = TVEC50_1 * EXP(ETA(1))
EC50_2 = TVEC50_2 * EXP(ETA(1))
EC50_3 = TVEC50_3 * EXP(ETA(1))

TVEC50_1_FENT = EXP(THETA(20))
TVEC50_2_FENT = TVEC50_1_FENT + EXP(THETA(21))
TVEC50_3_FENT = TVEC50_2_FENT + EXP(THETA(22))

EC50_1_FENT = TVEC50_1_FENT * EXP(ETA(3))
EC50_2_FENT = TVEC50_2_FENT * EXP(ETA(3))
EC50_3_FENT = TVEC50_3_FENT * EXP(ETA(3))

UA_1 = CE/EC50_1
```

```
UA_2 = CE/EC50_2

UA_3 = CE/EC50_3


UB_1 = 0

UB_2 = 0

UB_3 = 0

IF(OPIOID.EQ.1.AND.CE_FENT.GT.0) UB_1 = CE_FENT/EC50_1_FENT

IF(OPIOID.EQ.1.AND.CE_FENT.GT.0) UB_2 = CE_FENT/EC50_2_FENT

IF(OPIOID.EQ.1.AND.CE_FENT.GT.0) UB_3 = CE_FENT/EC50_3_FENT


U50_1 = 1

U50_2 = 1

U50_3 = 1


IF(OPIOID.EQ.1.AND.CE_FENT.GT.0) U50_1 = 1 - THETA(23) * UB_1 / (UA_1 + UB_1) + THETA(23) * (UB_1 / (UA_1 + UB_1))**2

IF(OPIOID.EQ.1.AND.CE_FENT.GT.0) U50_2 = 1 - THETA(23) * UB_2 / (UA_2 + UB_2) + THETA(23) * (UB_2 / (UA_2 + UB_2))**2

IF(OPIOID.EQ.1.AND.CE_FENT.GT.0) U50_3 = 1 - THETA(23) * UB_3 / (UA_3 + UB_3) + THETA(23) * (UB_3 / (UA_3 + UB_3))**2


DRUG1 = 0

DRUG2 = 0

DRUG3 = 0

IF(UA_1.GT.0.OR.UB_1.GT.0) DRUG1 = ((UA_1+UB_1)/U50_1)**GAMMA / (1 + ((UA_1+UB_1)/U50_1)**GAMMA)

IF(UA_2.GT.0.OR.UB_2.GT.0) DRUG2 = ((UA_2+UB_2)/U50_2)**GAMMA / (1 + ((UA_2+UB_2)/U50_2)**GAMMA)

IF(UA_3.GT.0.OR.UB_3.GT.0) DRUG3 = ((UA_3+UB_3)/U50_3)**GAMMA / (1 + ((UA_3+UB_3)/U50_3)**GAMMA)
```

```
;Pr[Y>=4], Pr[Y>=3], Pr[Y>=2], etc.
PGE1 = DRUG1
PGE2 = DRUG2
PGE3 = DRUG3

;Pr[Y=0], Pr[Y=1], etc.
P0 = 1-PGE1
P1 = PGE1-PGE2
P2 = PGE2-PGE3
P3 = PGE3

IF(P0.LT.0) EXIT
IF(P1.LT.0) EXIT
IF(P2.LT.0) EXIT
IF(P3.LT.0) EXIT

DV0 = 0
DV1 = 0
DV2 = 0
DV3 = 0
IF(DVTY.EQ.3.AND.DV.EQ.0) DV0 = 1
IF(DVTY.EQ.3.AND.DV.EQ.1) DV1 = 1
IF(DVTY.EQ.3.AND.DV.EQ.2) DV2 = 1
IF(DVTY.EQ.3.AND.DV.EQ.3) DV3 = 1

IPRED  = 3*P3 + 2*P2 + 1*P1
Y      = DV0*P0 + DV1*P1 + DV2*P2 + DV3*P3

; For simulation purposes
```

```
IF(ICALL.EQ.4) THEN
CALL RANDOM(2,R)
IF(R.GT.PGE1)                SDV=0
IF(R.GT.PGE2.AND.R.LE.PGE1) SDV=1
IF(R.GT.PGE3.AND.R.LE.PGE2) SDV=2
IF(R.LE.PGE3)                SDV=3
ENDIF

$THETA
0.223  FIX  ; V1; L
2.38   FIX  ; V2; L
2.51   FIX  ; V3; L
-0.359 FIX  ; CL; L.min-1
0.519  FIX  ; Q2; L.min-1
-1.47  FIX  ; Q3; L.min-1
2.5    FIX  ; Vm; L
-2.69  FIX  ; CLm; L.min-1
1.38   FIX  ; MTT; min
-0.888 FIX  ; Prop.error; ...
0      FIX  ; Add.error; ng/mL-1 ;; SD = 1 ng.mL-1
-1.74  FIX  ; Prop.error_met; ...
3.13   FIX  ; Add.error_met; ng/mL-1
(-4, -1.5, 1) ; ke0; min-1         ;;llp
(-1, 1.3, 3)    ; gamma; ...       ;;llp
5.4   ; C50_1; ng.mL-1             ;;llp
5.4   ; Delta_12; ng.mL-1          ;;llp
5.8   ; Delta_23; ng.mL-1          ;;llp
(-4, -2.1, 1) ; ke0_FENT           ;;llp
-0.5 ; C50_1; ng.mL                ;;llp
```

```
0.5  ; C50_2; ng.mL              ;;llp
1.4  ; C50_3; ng.mL              ;;llp
0 FIX ; INT; rel.

$OMEGA
0.1     ; BSV_C50                ;;llp
1E-2 FIX ; BSV_Gamma
1E-2 FIX ; BSV_C50_FENT

;$SIGMA
;1 FIX

$EST METHOD=CONDITIONAL LAPLACE SLOW LIKE NUMERICAL PRINT=1 SIG=3 SIGL=9 MAX=9999
NOABORT
$COV PRINT=E
```
